# Vaccination with Single Chain Antigen Receptors for Islet-Derived Peptides Presented on I-A^g7^ Delays Diabetes in NOD Mice by Inducing Anergy in Self-Reactive T-Cells

**DOI:** 10.1371/journal.pone.0069464

**Published:** 2013-07-24

**Authors:** Werner Gurr, Margaret Shaw, Raimund I. Herzog, Yanxia Li, Robert Sherwin

**Affiliations:** Department of Internal Medicine, Yale University, School of Medicine, New Haven, Connecticut, United States of America; Université Paris Descartes, France

## Abstract

To develop a vaccination approach for prevention of type 1 diabetes (T1D) that selectively attenuates self-reactive T-cells targeting specific autoantigens, we selected phage-displayed single chain antigen receptor libraries for clones binding to a complex of the NOD classII MHC I-A^g7^ and epitopes derived from the islet autoantigen RegII. Libraries were generated from B-cell receptor repertoires of classII-mismatched mice immunized with RegII-pulsed NOD antigen presenting cells or from T-cell receptor repertoires in pancreatic lymph nodes of NOD mice. Both approaches yielded clones recognizing a RegII-derived epitope in the context of I-A^g7^, which activated autoreactive CD4^+^ T-cells. A receptor with different specificity was obtained by converting the BDC2.5 TCR into single chain form. B- but not T-cells from donors vaccinated with the clones transferred protection from diabetes to NOD-SCID recipients if the specificity of the diabetes inducer cell and the single chain receptor were matched. B-cells and antibodies from donors vaccinated with the BDC2.5 single chain receptor induced a state of profound anergy in T-cells of BDC2.5 TCR transgenic NOD recipients while B-cells from donors vaccinated with a single chain receptor specific for I-A^g7^ RegII peptide complexes induced only partial non-responsiveness. Vaccination of normal NOD mice with receptors recognizing I-A^g7^ RegII peptide complexes or with the BDC2.5 single chain receptor delayed onset of T1D. Thus anti-idiotypic vaccination can be successfully applied to T1D with vaccines either generated from self-reactive T-cell clones or derived from antigen receptor libraries.

## Introduction

A therapy for type 1 diabetes that specifically attenuates self-reactive T-cells might reduce the potential for unwanted side effects inherent in non-specific approaches. Anti-idiotypic vaccination, in which the variable regions of antigen receptors act as vaccines, represents one such selective therapeutic approach. This type of vaccination has been used for lymphoma treatment to achieve targeting of cancer cells [1]; [2]. Applied to a T-cell-mediated autoimmune disease, the antigen recognized by the anti-idiotypic vaccine is a complex of MHC with a peptide derived from an autoantigen (pMHC complex). Vaccinations that involve anti-idiotypic responses have been tested with some success in experimental autoimmune encephalomyelitis and multiple sclerosis [3]–[7] as well as in other autoimmune diseases or models thereof [8]–[10]. However, apart from an early report on vaccination with heat shock protein 60 specific CD4^+^ T-cells in NOD mice [11], this vaccination approach has to date not been applied to type 1 diabetes.

Practically, development of an anti-idiotypic vaccine necessitates the generation of an autoreactive T-cell clone or a highly specific T-cell line. The anti-idiotypic immune response is then induced by vaccinating either with the autoreactive T-cell (T-cell vaccination), or the recombinant variable region or peptides corresponding to the complementary determining region (CDR) of the antigen receptor. In the current study we utilized the V-regions provided by the islet-reactive CD4^+^ T-cell clone BDC2.5, which were converted into a single chain receptor to yield the vaccine antigen. In addition we tested a novel approach for vaccine generation by constructing and selecting phage-displayed single chain antigen receptor libraries (single chain fragment variable, scFvs) for clones binding to a complex of MHC and a self-antigen-derived peptide. This overcomes the need for generation of self-reactive T-cell clones and lines, which may not always be possible, and establishes scFv libraries as permanent repositories of antigen receptor variable regions for the isolation of new anti-idiotypic vaccines. This approach to vaccine generation defines ‘idiotype’ as the entirety of the structure of an antigen receptor that is necessary to confer its antigenic specificity. An idiotype is built from the variable regions of a T-cell or a B-cell receptor (BCR or TCR V-regions). *In vivo* idiotypes are ‘displayed’ on T or B-cells, whereas in the approach taken here they are phage-displayed. Through the interaction with a peptide MHC complex a non-selected idiotype repertoire is shaped. *In vivo* and for T-cells this process occurs in the thymus where specialized antigen presenting cells (APCs) provide the pMHC complex. For the phage-displayed repertoire generated here, this process occurs *in vitro* on APCs taken from the spleen and pulsed with a desired antigen thus allowing manipulation of the process of repertoire selection.

To choose a relevant MHC-peptide complex we relied on previous studies indicating that the Reg proteins that have been associated with islet regeneration, and specifically RegII, might act as autoantigens in type 1 diabetes [12]; [13]. We observed that vaccination with the N-terminal fragment of RegII (NtfrRII) accelerated diabetes in NOD mice and that CD4^+^ T-cells from immunized mice transferred the disease to NOD-SCID recipients. RegII and specifically its N-terminal fragment (NtfrRII) presented in the context of the NOD MHC allele I-A^g7^, as well as the self-antigen chromogranin A (ChgA) recognized by the I-A^g7^-restricted BDC2.5 TCR of an islet-reactive T-cell clone [14]; [15] acted as model autoantigens to investigate whether the principle of anti-idiotypic vaccination could be applied to type 1 diabetes and whether vaccines could be generated based on the considerations outlined.

## Materials and Methods

### Ethics Statement

Experiments were approved by the Yale University’s Institutional Animal Care and Use Committee (IACUC). Protocol number 07349, “*Autoimmune Mechanisms of Type 1 Diabetes*”.

### Animals

Female NOD, NOD-SCID, B10.HTG H-2^g^ and BDC2.5 TCR transgenic NOD mice were obtained from Jackson Lab. Mice were kept under specific pathogen-free conditions on a 12–12 hrs daylight cycle and fed with autoclaved standard chow.

### Clinical Evaluation

Mice were tested for glucosuria with reagent strips for urinalysis (Bayer) and considered diabetic after two consecutive maximum readings of the color scale.

### Vaccinations

Mice where immunized either subcutaneously or intraperitoneally depending on the experiment. The adjuvant was alum for all vaccinations except for generation of the B-cell receptor repertoire for library construction (see below).

### B- and T-cell Receptor (BCR and TCR) Repertoires used for Library Construction

To generate BCR libraries, I-A^g7^-mismatched (B10.HTG H-2^g^) mice were immunized with NtfrRII-pulsed, glutaraldehyde-fixed spleen cells from NOD mice (total of three immunizations, the first in Complete Freund’s Adjuvant, the second and third in Incomplete Freund’s Adjuvant, 2×10^7^ cells per immunization). The TCR repertoire was derived from pancreatic lymph nodes of non-immunized NOD mice.

### Cloning of scFv Libraries from B-cells (BscFv)

scFv libraries were cloned in pAK, a phagemid that allows display of the scFvs as described by Krebber et al. [16]. This reference also contains a list of the appropriately tailed primers for cloning of V_h_ and V_l_ regions, a description of the individual cloning steps as well as protocols for phage rescue and precipitation, growth media for bacteria and induction conditions for expression of recombinant scFvs.

### Cloning of scFv Libraries from T-cells (TscFv)

TscFv libraries were cloned in the form Vα-(Gly_4_Ser_1_)_4_-Vβ. N-terminal sequence primers for the α and β chains were derived from Arden S et al. [17]. Two C-terminal primers were designed to anneal to a constant region of the TCR immediately adjacent to the V-region of the α- or β-chain. Primers (Tables S1–S5 in File S4) were tailed for scFv assembly and cloning into pAK according to Krebber at al. [16] (Fig. S2 in File S2). The size of TscFv libraries was 10^5^–2×10^6^ clones. >70% of clones contained SfiI inserts.

### Library Selection

Single chain V-region libraries were phage-displayed because this technology allows the selection of idiotypes with a desired specificity. Two figures are provided describing important features of this technology and details of the library selection procedure (Fig. S1A and S1B in File S1) [18]; [19]. Phage-displayed scFvs were first positively selected for binding to NOD spleen cells that had been pulsed with the N-terminal fragment of RegII (NtfrRII). Spleen cells were incubated for 51 hrs with IFN-γ (4 ng/ml) and received two pulses of NtfrRII (10 µg/ml) 12 and 3 hrs before library selection. Cells were washed and then incubated with the scFv library (1 hr in PBS with 3% BSA; 5×10^7^ spleen cells and 10^9^ to 10^10^ phages). After extensive washing, bound phages were eluted with 0.1 M glycine buffer pH 3. The eluate was neutralized with 1 M Tris pH 7.5 and phages were negatively selected on unpulsed spleen cells. Phages remaining in the supernatant of the second selection step were used to infect XL-1blue bacteria. Library selections were performed for three rounds for BscFvs and for four rounds for TscFvs.

### Library Screening

A) Clones of the final selection round were screened by FACS analysis. Phage precipitates were produced and used as primary antibody to stain NtfrRII-pulsed and unpulsed spleen cells (5×10^8^–5×10^9^ phages/10^6^ cells). Bound phages were detected with a biotinylated anti M13 antibody (Abcam) followed by streptavidin FITC (Sigma).

B) Clones were grown in *E. coli* strain HB2151 overnight (deep-well plates, Nunc) followed by induction of protein expression. Bacteria were lysed in 400 µl 0.5× cell lysis reagent (Cell Lytic B, Sigma) diluted with 40 mM Tris buffer pH 8.2 and protease inhibitors. The supernatant was loaded to highQ anion exchange medium (Vivawell Q-IEX protein purification plate). After two wash steps, bound protein was eluted (40 mM Tris, pH 7.2 and 1 M NaCl) and anti c-myc agarose was added (40 µl/well, Sigma). After incubation, the resin was washed and bound scFvs were eluted (0.1 M glycine buffer pH 3). The eluate was neutralized, dialyzed against 40 mM ammonium bicarbonate (DispoDialyzer plate, Cole-Parmer) and freeze-dried. ScFvs were dissolved directly in tissue culture medium (Bruff’s, Gibco/Invitrogen) and tested in proliferation and IL-2 ELISPOT assays with lymphocytes from draining lymph nodes of NtfrRII-immunized, CD8^+^ T-cell-depleted, NOD mice.

### Generation of the BDC2.5 TscFv

mRNA coding for the BDC2.5 α and β chains was isolated from CD4^+^ T-cells of BDC2.5 TCR transgenic mice. cDNA was amplified with the constant region primers used for the generation of the TscFv libraries and the appropriate N-terminal Vα and Vβ primers. The fully assembled BDC2.5 TscFv was cloned into and expressed from pAK.

### Peptide Synthesis

Two sets of peptides were synthesized by the Yale Small Scale Peptide Synthesis facility. Set S covered NtfrRII (from amino acid position 23 to 75) by 19 peptides. Peptides of set S were combined into 9 peptide pools, each pool containing 2 overlapping peptides (the last pool contained 3 peptides). Set L covered NtfrRII by four overlapping peptides. (The sequences of the peptides contained in set S and set L are provided in Table S6 in File S4).

### T-cell Transfer to NOD-SCID Mice

Inducer cells were obtained from spleen cells of NOD mice immunized with NtfrRII (three immunizations i.p. at 4, 7 and 11 weeks of age (30 µg/mouse)) or from NOD mice transgenic for the BDC2.5 TCR. Test cells were obtained either by depletion of pan T-cells or B-cells from spleen cells of NOD mice immunized with scFv clones and control antigens or from non-immunized NOD mice. Depletion beads were from Dynal/Invitrogen (for T-cells) or from Qiagen (for B-cells). 6×10^6^ inducer cells (3×10^6^ for BDC2.5) and 1.8×10^7^ test cells were transferred per mouse. Test cells were prepared 10 days after the second immunization, suspended in PBS and 100 µl were injected retro-orbitally into NOD-SCID mice anesthetized with ketamine/xylazine (100 mg/kg). Inducer cells were transferred under anesthesia by the same route 24 hrs later.

### Structural Alignment of scFvs

A threading approach to protein structure prediction was employed to generate 3D protein models for BscFv D9 and D9mut and for TscFvs S9/P2 and BDC2.5. The approach uses known structures deposited in the protein data bank to predict tertiary conformations of a given amino acid sequence [20]; [21].

## Results

**Figure 1:**
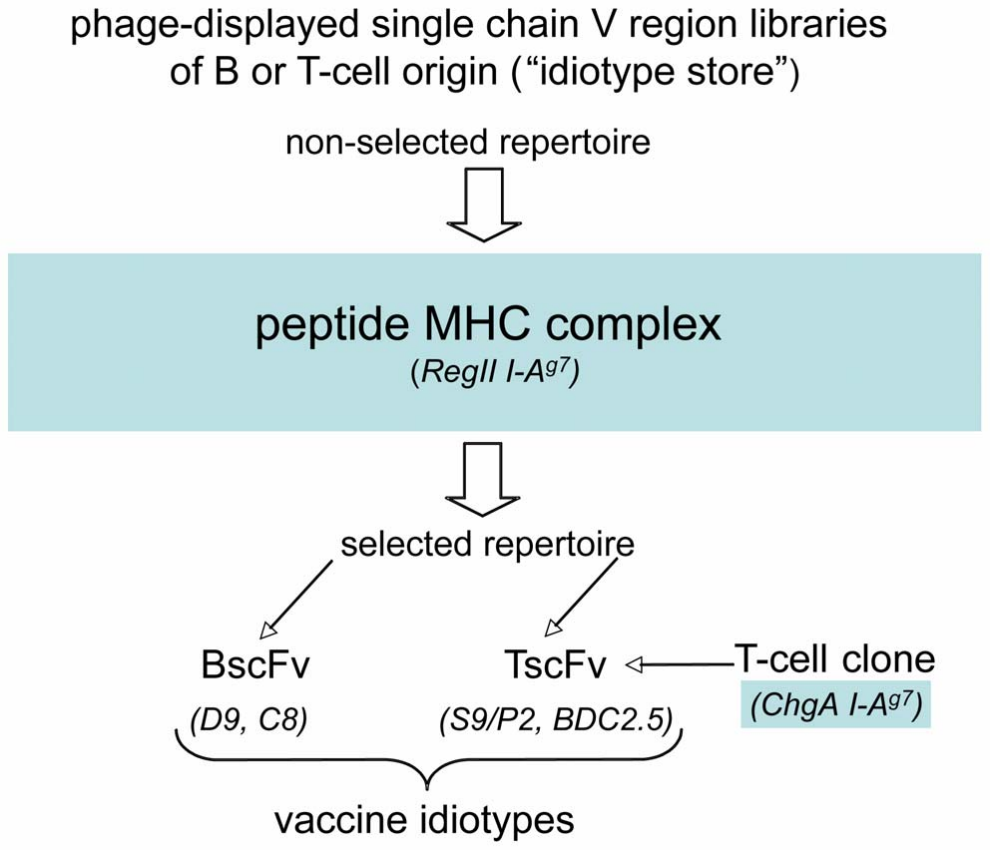
Considerations for the generation of an anti-idiotypic vaccine. a) The peptide MHC (pMHC) complex determines the structure of the idiotype. In this report two pMHC complexes are employed: a) RegII and b) chromogranin A (ChgA)-derived epitopes presented in the context of the NOD classII MHC I-A^g7^. Both pMHC complexes are ‘diabetogenic’ i.e. they stimulate autoagressive T-cells that target islets. b) Regardless of its origin an idiotype is a suitable vaccine if it binds the pMHC complex recognized by the targeted T-cell receptor. Consequently, an idiotype represented by BCR V-regions recognizing a given pMHC complex may be a suitable vaccine capable of inducing an anti-idiotypic B-cell response, able to cross-react with a TCR recognizing the same pMHC complex. Thus cross-reactivity based on structural similarities between idiotypes is required. B-cells (immunoglobulins) can fulfill this requirement and their involvement in mediating the vaccine effects ought to be expected. Furthermore, the effect of the vaccination should be reduced if a vaccine idiotype is not matched to the idiotype represented by the TCR of the targeted T-cell (i.e. if the two idiotypes recognize a different pMHC complex). To investigate these implications experimentally we required a) two structurally similar idiotypes with different origin i.e. two idiotypes recognizing the same pMHC complex, but with different primary sequences and b) two structurally dissimilar idiotypes i.e. each recognizing a different pMHC complex. These requirements guided the process of vaccine generation. c) Practically, an idiotype may either be selected - on a given pMHC complex - from a phage-displayed single chain variable region library of B-cell or T-cell origin (libraries correspond to “idiotype repositories” or “stores”) or it may be derived from an existing T-cell clone. Clones D9 and C8 are BCR V-region-derived clones while S9/P2 and BDC2.5 are TCR V-region-derived clones. To denote the origin of a BCR V-region-derived clone we use the term BscFv. A BscFv has the form V_light chain_-linker-V_heavy chain_. For a TCR V-region-derived clone we use the term TscFv. A TscFv has the form V_α chain_-linker-V_β chain_. The BscFv clones D9, C8 and the TscFv clone S9/P2 recognize I-A^g7^ complexed with a RegII (NtfrRII)-derived epitope. The BDC2.5 V-region single chain clone (TscFv) is taken from the TCR of a diabetogenic T-cell that recognizes chromogranin A (ChgA)-derived epitopes in the context of I-A^g7^ (more information on BDC2.5 is given later).

This section contains two main components. The first component deals with the acquisition and characterization of the scFvs (idiotypes) used for vaccination (BscFvs D9, C8 and TscFvs S9/P2 and BDC2.5, see Fig. 1) and describes the application of the phage-displayed BscFv D9 to determine the temporal dynamics of a ‘diabetogenic’ pMHC complex in pancreatic lymph nodes of NOD mice. The second part of the report contains results on the vaccinations and the mechanism underlying the observed effects.

### 1. Vaccine Generation

#### 1.1 BscFv clones D9 and C8

*Clones recognizing I-A^g7^ NtfrRII peptide complexes isolated from scFv libraries generated from the B-cell receptor V-region repertoire identify a T-cell epitope for autoaggressive CD4^+^ T-cells.*

**Figure 2:**
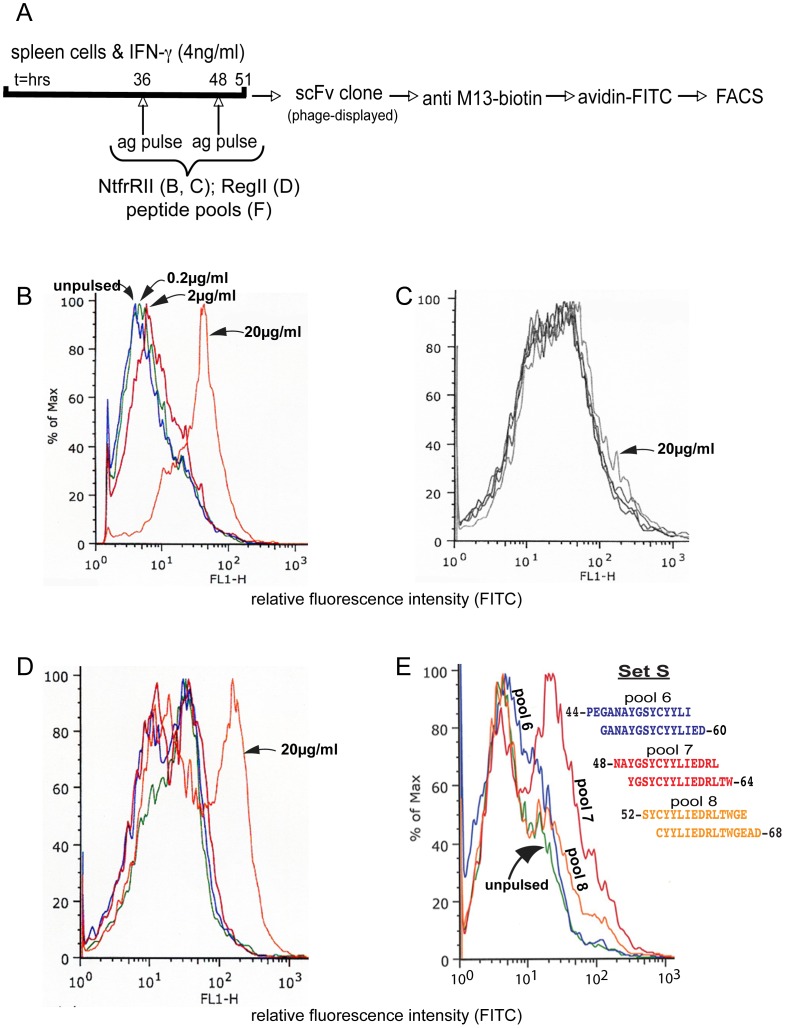
BscFv clone D9, an idiotype that binds I-A^g7^ NtfrRII peptide complexes and identifies an NtfrRII-derived T-cell epitope. scFv clones were characterized to establish that they represent the desired idiotype. Here the idiotype should recognize an NtfrRII (RegII)-derived epitope presented in the context of I-A^g7^ but it should not bind to the surface of spleen cells presenting NtfrRII-derived epitopes in the context of other classII MHC alleles. BscFv clone D9 was used as primary antibody to stain antigen-pulsed and unpulsed spleen cells according to the protocol shown in A. IFN-γ was added to the spleen cells to enhance expression of I-A^g7^. Spleen cells of NOD mice were pulsed with NtfrRII at the indicated concentrations and stained with D9 (B). Spleen cells of B10 HTG H-2^g^ mice (K^d^/D^b^/I-A^d^/I-E^d^) expressing different classII MHC alleles but the same classI alleles as NOD mice (K^d^/D^b^/I-A^g7^) pulsed with the same doses of NtfrRII failed to bind D9 (C). Spleen cells of NOD mice pulsed with full-length RegII also bound D9 at the highest pulse dose (D). The staining protocol on NtfrII-pulsed vs. unpulsed NOD APCs (B) was performed routinely to characterize newly produced batches of phage-displayed scFvs and the graph shown is representative of one of the batches. Pulse conditions used in (C) and (D) were tested with three different batches of D9 (representative graphs are shown). In order to map the NtfrRII-derived epitope recognized by D9 in the context of I-A^g7^, NOD spleen cells were pulsed with pools each consisting of two overlapping peptides of set S (Table S6 in File S4) covering NtfrRII. They were stained with D9 and analyzed by flow cytometry. D9 preferentially bound to APCs pulsed with pool 7 (peptides 48–62 and 50–64) and weakly to APCs pulsed with pool 6 (peptides 44–58 and 46–60) or pool 8 (peptides 52–66 and 54–68) (E). Spleen cells pulsed with peptide pools outside the region 44–68 did not stain with D9. The graph (E) is representative of three independent staining runs performed on NOD spleen-cells pulsed with the peptide pools 6 or 7 or 8 of set S.

An scFv library was generated from the B-cell receptor variable region repertoire of classII-mismatched mice that had been immunized with NtfrRII-pulsed NOD antigen presenting cells. FACS analysis of individual phage-displayed scFv clones from the selected repertoire on NtfrRII-pulsed vs. unpulsed NOD APCs (Fig. 2A) identified clone D9. D9 recognized NtfrRII-pulsed NOD spleen cells with staining intensities dependent on the strength of the antigen pulse given (Fig. 2B). D9 failed to bind to spleen cells from NtfrRII-pulsed classII-mismatched mice (B10HTG H-2^g^) indicating that it recognized a peptide I-A^g7^complex and not NtfrRII bound to the cell surface or a peptide classI (K^d^ or D^b^) complex (Fig. 2C). D9 also recognized NOD spleen cells that had been pulsed with the full length RegII protein indicating that the peptide recognized in the context of I-A^g7^ was not a cryptic T-cell epitope (Fig. 2D). A form of D9 lacking part of its V_h_ chain (termed D9mut) was also generated that failed to bind to NtfrRII-pulsed spleen cells (data not shown).

We previously reported that NtfrRII must contain at least one epitope for I-A^g7^-restricted, self-reactive T-cells, because CD4^+^ T-cells from donors vaccinated with NtfrRII transfer disease to NOD-SCID recipients. An scFv selected on NtfrRII-pulsed APCs should be able to identify the location within NtfrRII of such an epitope. NOD spleen cells were therefore pulsed with pools of peptide set S covering NtfrRII (Table S6 in File S4) and then analyzed by FACS with D9 as primary antibody. While spleen cells pulsed with pools 1–5 containing peptides covering amino acids 22–56 and pool 9 containing peptides covering amino acids 56–75 of NtfrRII did not stain with D9, those pulsed with pools 6–8 yielded positive staining (Fig. 2E). Peptide pool 7 (NtfrRII 48–64) produced the strongest staining and identified this region as the location of a potential epitope for CD4^+^ T-cells.

**Figure 3:**
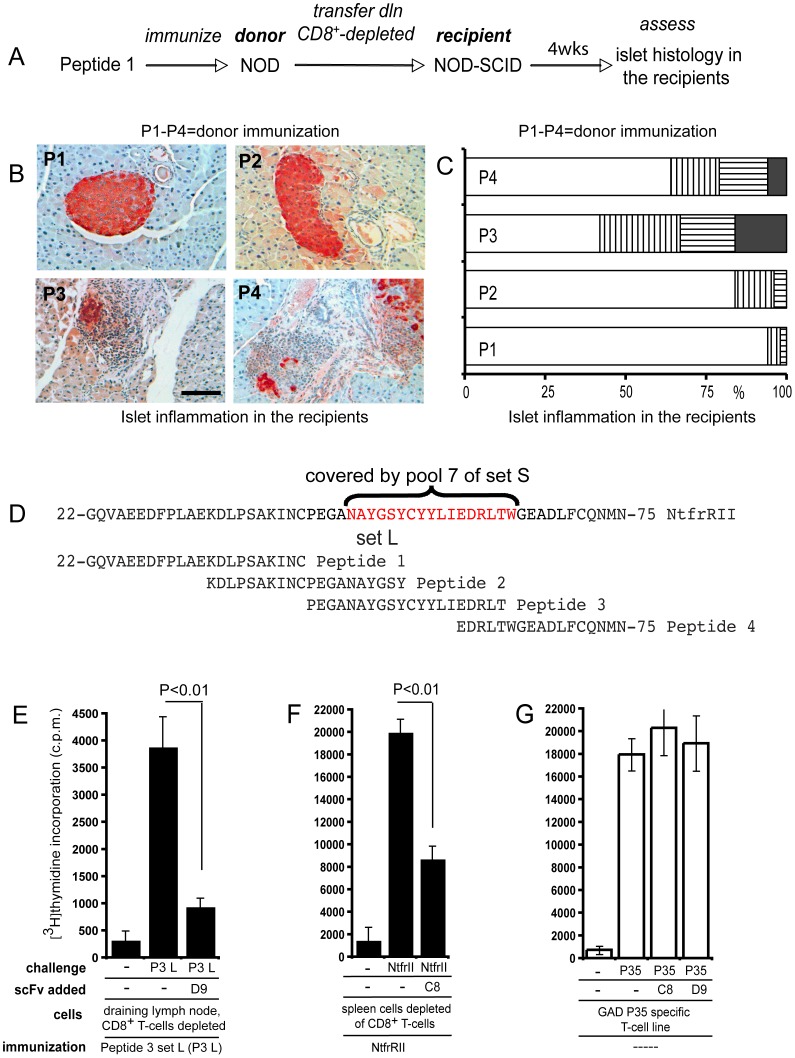
Autoreactive CD4^+^ T-cells and BscFv D9 recognize the same I-A^g7^ NtfrRII peptide complex. Four groups of NOD mice were immunized s.c. with the four peptides of set L in alum, one peptide for each group of mice. Set L covers NtfrII and by applying the procedure shown for peptide 1 (A) to the remaining peptides in set L the ability of each peptide to activate NtfrRII specific CD4^+^ islet self-reactive T-cells could be assessed. Set L Peptide 1 (P1) 22-GQVAEEDFPLAEKDLPSAKINC-43 Peptide 2 (P2) 34-KDLPSAKINCPEGANAYGSY-53 Peptide 3 (P3) 44-PEGANAYGSYCYYLIEDRLT-63 Peptide 4 (P4) 59-EDRLTWGEADLFCQNMN-75 This was accomplished by transferring CD4^+^ T-cells from the vaccinated NOD donor mice to NOD-SCID recipients and then - four weeks after transfer - studying the islet histology in the NOD-SCID recipients. Few infiltrated islets existed in recipients of CD4^+^ T-cells derived from P1- and P2-vaccinated donors. In contrast, as shown in B, extensive infiltration with islet destruction was observed in recipients of CD4^+^ T-cells from P3- and P4-vaccinated donors (scale bar: 50 µm). For each peptide a total of 60–80 islets from pancreata of three recipients were assessed for islet inflammation (C) (white bars: no infiltration; vertically striped bars: peri islet infiltration; horizontally striped bars: intra islet infiltration; black bars: islet destruction). Figure 3D demonstrates that the region of NtfrRII covered by peptide pool 7 of set S (RegII 48–64, as indicated in red), which was preferentially bound by BscFv D9 when presented in the context of I-A^g7^, overlaps to a large extent with peptide 3 of set L (RegII 44–63). Since peptide 3 specific T-cells infiltrate and destroy islets it is highly likely that RegII specific autoaggressive CD4^+^ T-cells exist in the NOD mouse that recognize the same I-A^g7^ RegII peptide complex as does BscFv D9. To test this possibility further we examined if recombinant D9 could attenuate the proliferation of CD4^+^ T-cells from peptide 3 set L-vaccinated NOD mice. For this purpose recombinant clone D9 (10 µg/ml) was added to a proliferation assay of spleen cells depleted of CD8^+^ T-cells from peptide 3 set L-vaccinated NOD mice (E). A second BscFv clone, termed C8 that suppressed proliferation of NtfrRII specific CD4^+^ T-cells was tested with a T-cell assay similar to that used to test D9. However, in this case donors were vaccinated with the full-length NtfrRII given that the epitope recognized by C8 in the context of I-A^g7^ had not been mapped (F). To determine whether the attenuation of proliferation was antigen specific we used a previously established CD4^+^ T-cell line, which recognizes peptide 35 of the autoantigen glutamic acid decarboxylase (GAD) in the context of I-A^g7^. GAD peptide 35 (524-SRLSKVAPVIKARMMEYGTT-543) is one of several epitopes identified in this protein that can activate NOD self-reactive CD4^+^ T-cells. GAD peptide 35 presented in the context of I-A^g7^ should not be recognized by either C8 or D9, which are idiotypes binding to RegII (NtfrRII)-derived epitopes presented in the context of I-A^g7^. Neither C8 nor D9 attenuated proliferation of the GAD peptide 35 specific T-cell line (G). Statistical evaluation was performed by t-test. Representative graphs are shown in E, F and G; three independent experiments were performed. For T-cell proliferation assays in this report triplicates were counted.

We next determined whether NOD mice have autoreactive CD4^+^ T-cells activated by the epitope contained within the 44–68 region of NtfrRII that is recognized by D9 in the context of I-A^g7^. For this purpose, NOD donors were immunized with peptides of set L covering NtfrRII. CD4^+^ T-cells were obtained from the draining lymph nodes and were transferred to NOD-SCID recipients to test if they infiltrated and destroyed the islets of the recipients (Fig. 3A). The degree of infiltration and islet destruction observed in the recipients depended on the peptide used to vaccinate the donors. Recipients of CD4^+^ T-cells from donors vaccinated with peptides 1 or 2 (RegII amino acid residues 22–53) developed only mild islet infiltration and no islet destruction while recipients of T-cells from donors vaccinated with peptides 3 and 4 (RegII amino acid residues 44–74) developed more pronounced infiltrates with islet destruction (Fig. 3B and C). These observations and the extent of the overlap (Fig. 3D) between peptide 3 of set L (amino acids 44–63) and pool 7 of set S (amino acids 48–64) preferentially recognized by D9 in the context of I-A^g7^ suggested that, in NOD mice, region 48–64 of RegII contains an epitope recognized by D9 and by autoreactive CD4^+^ T-cells with islet destructive potential.

The pAK system allows production of the recombinant (as opposed to phage-displayed) form of scFvs. Since vaccination with peptide 3 of set L could stimulate self-reactive T-cells and the activating pMHC complex was recognized by D9, we tested whether recombinant D9 could attenuate the proliferation of CD4^+^ T-cells from peptide 3 set L-vaccinated mice. Consistent with the epitope mapping performed previously, addition of recombinant clone D9 was able to reduce the rate of proliferation of T-cells specific for the RegII peptide 44–63 (Fig. 3E).

While D9 had been isolated from the selected BscFv library by FACS analysis on NtfrRII-pulsed vs. unpulsed NOD APCs, we had also tested a different screening approach based on the ability of individual recombinant BscFv clones to reduce proliferation of NtfrRII-specific CD4^+^ T-cells. This screening approach identified a second BscFv clone termed C8 that was able to suppress proliferation of NtfrRII specific CD4^+^ T-cells (Fig. 3F). To test if the attenuation of proliferation by the BscFv clones D9 and C8 was antigen specific, we used NOD CD4^+^ T-cells that recognized a pMHC complex arising from the presentation of an epitope derived from an antigen different from NtfrRII (RegII). This complex should not be recognized by D9 and C8 and consequently the BscFvs should not affect the stimulation of T-cells with this specificity. Glutamic acid decarboxylase (GAD) is a well-studied islet self-antigen in T1D [22] and several T-cell epitopes in this protein have been identified. We had previously generated a CD4^+^ T-cell that recognized one these GAD epitopes termed peptide 35 (GAD 524–543) [23]. This line was used to test whether the attenuation mediated by D9 and C8 was dependent on the presence of NtfrRII (RegII) I-A^g7^ pMHC complexes and therefore antigen specific. Neither BscFv clone D9 nor C8 blocked the proliferation of this T-cell line (Fig. 3G). (Sequence data of D9, D9mut and C8, are provided in Tables S7 and S8 in File S4).

#### 1.2 An application for phage-displayed BscFv D9

*The number of I-A^g7^ NtfrRII peptide complexes in the pancreatic lymph nodes of NOD mice exhibits a biphasic pattern, followed, with an offset of two weeks, by the corresponding T-cell responses.*

**Figure 4:**
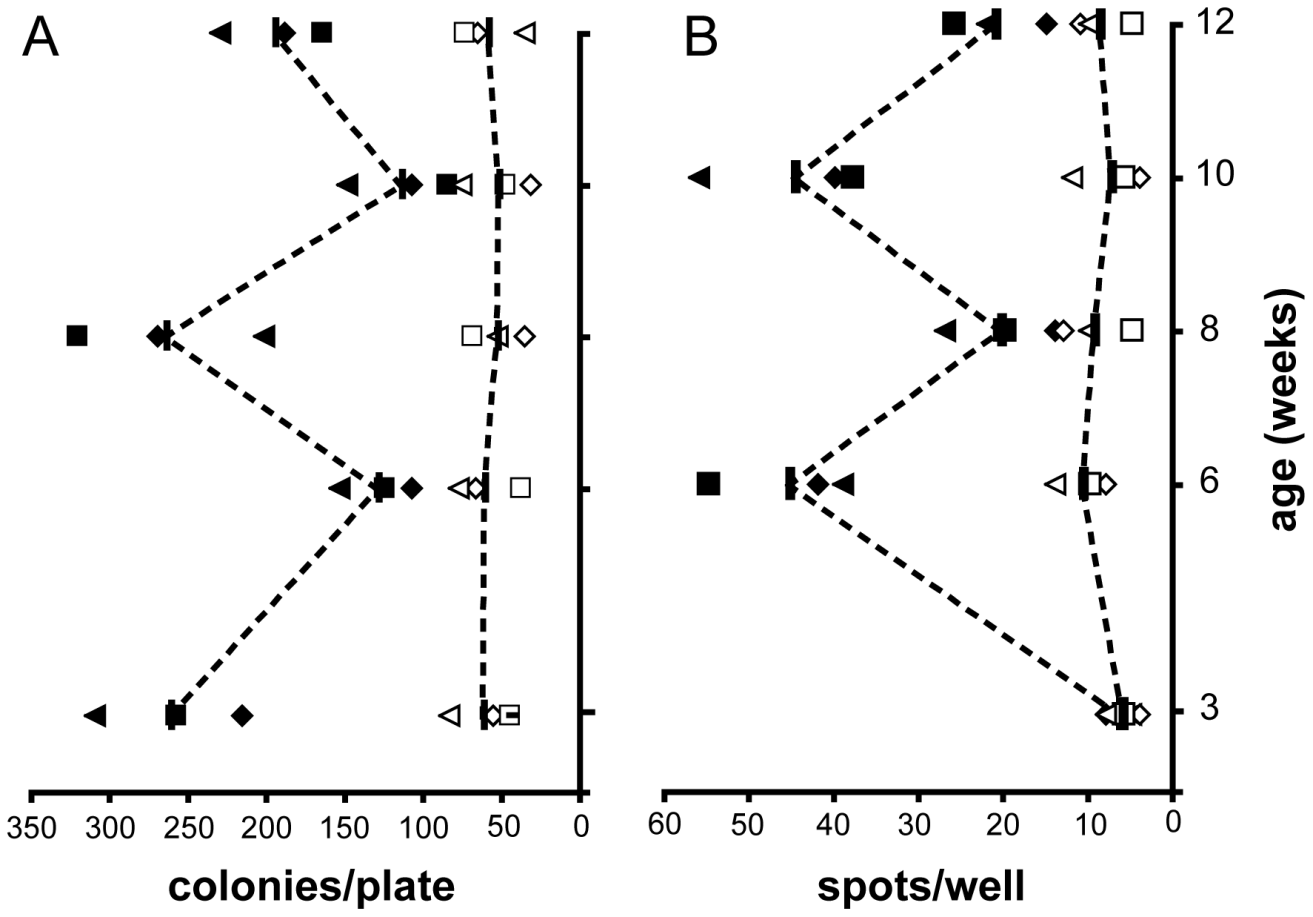
Application for phage-displayed BscFv D9. To obtain the temporal profile of the RegII 48–64 I-A^g7^ complex in NOD pancreatic lymph nodes (pln) binding of phage-displayed D9 (which recognizes this pMHC complex) was quantified (A). 10^6^ cells from pancreatic lymph nodes collected at the indicated time points were incubated with 10^9^ phages either displaying scFv D9 or the non-binding scFv D9mut, the latter being included as reference. After extensive washing, bound phages were eluted at low pH and used to infect XL-1blue bacteria. Phage infection transfers resistance to chloramphenicol (the chloramphenicol resistance gene being incorporated into the pAK system used here), which allows the bacteria to grow on chloramphenicol agar plates. The number of bacterial colonies obtained is proportional to the number of phages in the low pH eluate. For each experiment, eluted D9mut phages were first diluted aiming for a target count of 50 colonies per plate. The same dilution was then applied to the eluted D9 phages. The Y-axis units in (A) are colonies/plate. This number is proportional to the number of phages in the eluate obtained after incubating pln cells either with phage-displayed D9 (black symbols) or with D9mut (white symbols). To correlate the NtfrRII 48–64 I-A^g7^ pMHC complex profile obtained in (A) with the spontaneous NtfrRII T-cell response in the pancreatic lymph nodes we used an ELISPOT assay to detect IL-2 released from activated T-cells. Single cell suspensions of pln collected at the indicated ages were incubated with NtfrRII at 2.5 µg/ml or remained without antigen for 48 hrs. They were then transferred to an ELISPOT plate and incubated for 24 hrs to detect IL-2 releasing cells (2.5×10^5^ pln cells/well). The Y-axis in (B) shows the number of spots obtained from pln cells incubated with NtfrRII (black symbols) or from pln cells incubated without antigen (white symbols). T-cell response and I-A^g7^ Reg 44–68 pMHC complex profiles were each obtained from three sets of NOD mice with each set consisting of mice at 3, 6, 8, 10 and 12 weeks of age (four mice per time point). Each black or white symbol represents the colony or spot count from one set of mice. Both profiles are biphasic with the T-cell profile following the pMHC complex profile with a delay of two weeks. The differences in the Y-values between two consecutive time points of profiles A and B are statistically significant (P<0.05; t-test) for the experimental profiles represented by black symbols (colonies/plate obtained from incubation with D9; spots obtained by incubation with NtfrRII).

The isolation of BscFv clone D9 provided a tool to measure I-A^g7^ complexed with a RegII-derived epitope between residues 48–64. This was used to generate a time course of these complexes in the pancreatic lymph nodes of NOD mice during the pathogenesis of T1D (Fig. 4A). The profile of the spontaneous anti NtfrRII T-cell response at this site was also determined (Fig. 4B) to assess whether these profiles would indicate the presence of a vicious RegII-driven cycle operating during the pathogenesis of T1D in NOD mice. If such a cycle occurs it might be expected to lead to a continuous increase of I-A^g7^ NtfrRII peptide complexes in the pancreatic lymph nodes during the pathogenesis of T1D, which would be accompanied (or followed) by a continuous increase in the number of NtfrRII specific T-cells. However, a biphasic pattern was observed instead with an initial peak of I-A^g7^ NtfrRII peptide complexes occurring at 3 weeks of age followed by a second peak at 8 weeks of age. Although NtfrRII specific CD4^+^ T-cell responses in the pln had the same biphasic pattern, it was shifted by two weeks, with the first peak at 6 weeks and a second at 10 weeks of age suggesting a temporally restricted operation of a vicious cycle occurring between 6 and 10 weeks of age.

#### 1.3 TscFv clones S9/P2 and BDC2.5

*Single chain libraries (TscFvs) can be generated from the T-cell receptor variable region repertoire in pancreatic lymph nodes of non-immunized mice and can be selected for recognition of I-A^g7^ NtfrRII peptide complexes.*

The generation of a single chain library derived from TCR V-regions provides an idiotype ‘store’ with primary sequences different from the library used to isolate D9, a BscFv. Applying an appropriate selection and screening method to a single chain TCR library could identify an idiotype similar to D9 but this time represented by a TscFv thus providing two idiotypes built from two different primary sequences but recognizing the same antigen (pMHC complex). This would allow structural comparison of the two idiotypes and testing if anti-idiotypic serum produced against one would precipitate the other. The rationale for these experiments arose from the considerations guiding the idiotype selection outlined in Fig. 1.

If a serum produced against a given idiotype can cross-react with a second idiotype of different primary sequence but similar structure (recognizing the same antigen) the origin and primary sequence of an idiotype is unlikely to be essential in order for it to be an efficient vaccine. What would be important is that an idiotype - be it derived from a BscFv or a TscFv library- recognize a pMHC complex activating self-reactive T-cells because the TCR of an actual T-cell would have the same idiotype or one sufficiently similar to allow a serum produced against a library-derived idiotype to cross react with the actual TCR. The TCR of an actual RegII specific T-cell was not available, but having two similar idiotypes built from different primary sequences allowed us to study a comparable situation.

**Figure 5:**
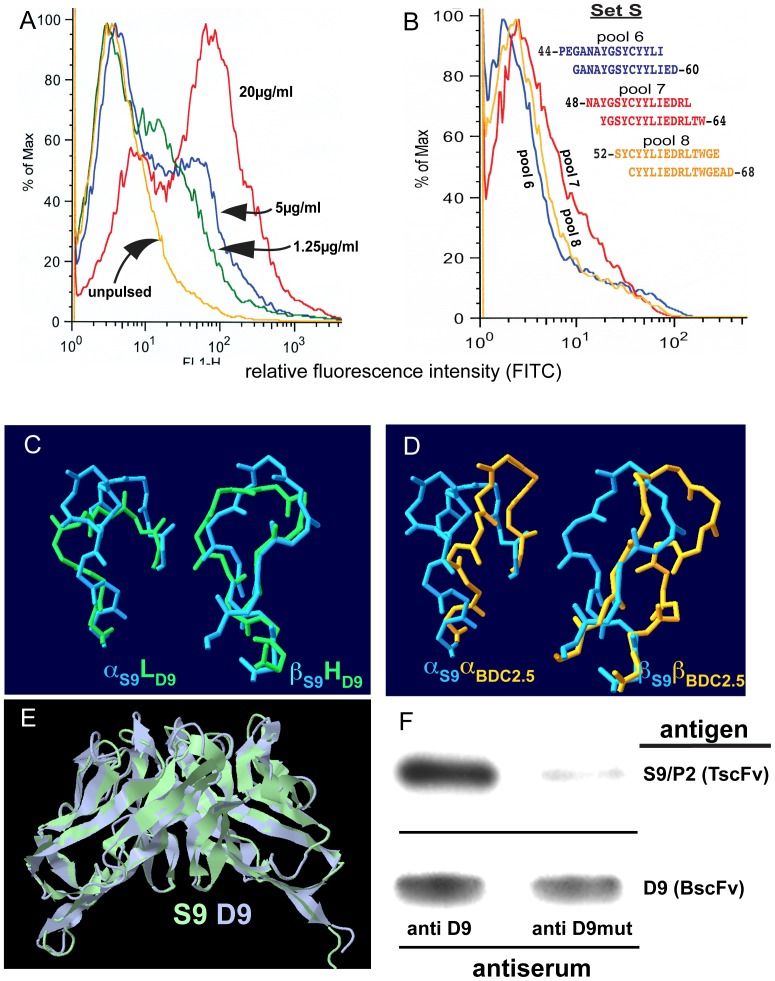
TscFv S9/P2, an idiotype structurally similar to that of BscFv D9. Given that D9 (a BscFv) is an idiotype with known specificity the aim was to isolate another idiotype structurally similar to D9, but different in its primary sequence. To achieve this aim a TscFv library was generated and selected by applying two different selection methods: 1) precipitation of the TscFv library with anti D9 antiserum that had been adsorbed with D9mut to reduce the presence of antibodies recognizing linear epitopes and 2) selection of the TscFv library on NtfrRII-pulsed NOD APCs. The criterion to screen the two resulting selected libraries P and S (same clone isolated by both selection methods) was chosen to yield an idiotype that recognized the same pMHC complex as D9. Spleen cells of NOD mice were pulsed with NtfrRII and stained with TscFv S9/P2 (A) or pulsed with NtfrRII peptide pools of set S (B). As with D9, spleen cells pulsed with peptide pools outside the region 44–68 of RegII did not stain with S9/P2. Given that BscFv D9 and TscFv S9/P2 represent two idiotypes with different primary sequences, but recognizing the same pMHC complex, we assessed their structural relatedness. A direct comparison of the CDR3 loops of D9 (green) with S9/P2 (blue) revealed structural overlap despite significant differences on the primary sequence level (Table S10 in File S4). The structural overlap was most notable for the β-chain of TscFv S9/P2 and the heavy chain of BscFv D9 (C). In contrast, a comparison of the CDR3 loops of TscFv S9/P2 (blue) and TscFv BDC2.5 (orange), which recognize different epitopes in the context of I-A^g7^, showed marked differences (D). The structural homology between BscFv D9 and TscFv S9/P2 extended beyond the CDR3 regions to encompass the entire molecule (idiotype) as shown in (E) and as evidenced by the template modeling (TM) scores for this alignment, which are provided in Table 1. In keeping with this observation, anti D9 serum was able to precipitate both BscFv D9 and TscFv S9/P2, anti D9mut serum precipitated D9, but only marginally TscFv S9/P2 (F). Lysates of bacteria expressing D9 or S9/P2 were precipitated with sera from anti D9 or anti D9mut-immunized mice. Precipitates were captured by immobilized protein A/G, were run in adjacent lanes of an SDS-PAGE gel and blotted to nitrocellulose. Bands were detected with an anti c-myc, HRP-labeled antibody. The bands corresponding to the scFvs run at an apparent molecular weight of ∼ 29 kD. The experiment was repeated once yielding the same result.

Autoreactive T-cells are concentrated in the pancreatic lymph nodes of NOD mice. We therefore generated a TscFv library from pln of 10-week old NOD mice, the age when peak proliferation of NtfrRII specific T-cells occurs (technical details are provided in Figure S2 in File S2 and Tables S1–S5 in File S4). This initial phage-displayed library was selected by two different methods. As with the previously described BscFv library, it was enriched on pulsed vs. unpulsed NOD spleen cells (4 rounds) yielding a selected (S) library. It was also precipitated with anti D9 immune serum yielding a precipitated (P) library. Sequence analysis of P and S libraries identified one clone, which had been obtained both by selection and precipitation, termed S9/P2 (the sequence of S9/P2 is provided in Table S9 in File S4). As with BscFv clone D9 that was used to generate the precipitating immune serum, TscFv S9/P2 bound NtfrRII-pulsed NOD APCs (Fig. 5A) but not classII-mismatched B10HTG H-2^g^ APCs (data not shown). Furthermore this clone also showed preferential binding to the NtfrRII T-cell epitope 48–64 in the context of I-A^g7^ identified by D9 (Fig. 5B). Primary sequence alignment (Table S10 in File S4) showed that there are no extended regions of sequence homology between TscFv S9/P2 and BscFv D9. However, upon generating and comparing 3D models for both scFvs, it was found that D9 and S9/P2, a BscFv and a TscFv recognizing the same peptide MHC complex, are more closely related than S9/P2 and BDC2.5, two TscFvs recognizing two different epitopes in the context of I-A^g7^ (Fig. 5C, D, E and Table 1). Accordingly, TscFv S9/P2 could be precipitated with serum obtained by vaccination with BscFv D9. In contrast, serum obtained by vaccination with BscFv D9mut, which has a disrupted idiotype and no longer recognized the I-A^g7^ RegII 48–64 pMHC complex, precipitated D9 but had little avidity for S9/P2 (Fig. 5F).

**Table 1:** Template modeling scores [TM] and root mean square deviation scores (RMSD) for the comparison of structural similarities between scFvs.

	TscFv		BscFv	
	S9/P2	BDC2.5	D9	D9mut
D9	[0.94133]; (1.22)	[0.83072]; (2.67)	[1]; (0)	[0.60388]; (1.96)
S9/P2	[1]; (0)	[0.85166]; (2.77)	–	–

The higher the template modeling score [TM] and the lower the root mean square deviation score (RMSD) the more closely two structures are related. Comparison of two identical structures (D9 and S9/P2 each with itself) yields 1 for the TM score and 0 for the RMSD score. The TM score weighting algorithm is less sensitive to distant than to close matches and is therefore a more sensitive measure for structural similarity than the RMSD score [46].

While D9, C8 and S9/P2 are library-derived scFvs it is possible - if autoaggressive T-cell clones are available - to derive a vaccine idiotype from such a clone by converting its TCR into an scFv. In T1D the autoaggressive clone BDC2.5 [24]–[26] has been investigated for years and NOD mice that are transgenic for its TCR are commercially available. Since such mice are essential for *in vivo* studies investigating the mechanism underlying anti-idiotypic vaccination we converted BDC2.5 into an scFv of the same form as S9/P2. Furthermore, an idiotype specific for a pMHC complex arising from the presentation of epitopes derived from an antigen different from NtfrRII (RegII) would allow us to address the specificity of the effects induced by vaccination. The BDC2.5 TCR recognizes two epitopes derived from chromogranin A, namely 29–42 and 358–371, when presented in the context of I-A^g7^ [14]; [15]. In addition it can also recognize - in the context of I-A^g7^- an array of mimotopes, designer peptides not derived from actual antigens that can stimulate BDC2.5 because they contain appropriately placed amino acid residues essential for the pMHC-TCR interaction [27]. Some of these mimotopes stimulate BDC2.5 more efficiently than its actual epitopes and thus could be used for studies involving T-cell proliferation.

**Figure 6:**
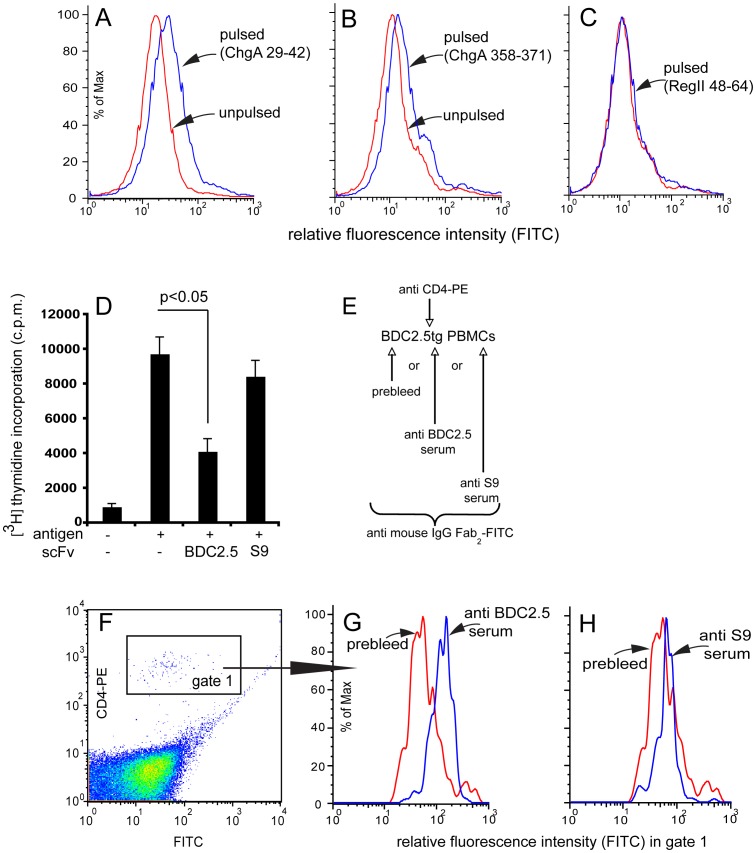
BDC2.5 TscFv, an idiotype structurally different from D9 and S9/P2. The availability of NOD mice transgenic for the TCR of the islet-autoreactive T-cell clone BDC2.5 provides a means to investigate *in vivo* effects induced by vaccination and, by generating an idiotype different from D9 and S9/P2, the specificity of the vaccination may be addressed. BDC2.5 TCR was therefore converted into a TscFv. Like the actual BDC2.5 TCR, BDC2.5 TscFv recognized NOD APCs pulsed with chromogranin A epitope 29–42 (24-DTKVMKCVLEVISD-42) (A) and NOD APCs pulsed with ChgA epitope 358–371 (358-WSRMDQLAKELTAE-371 (B). In contrast, NOD APCs pulsed with RegII epitope 48–64 were not recognized (C), thereby confirming that this process retains the idiotype of the original TCR. Staining of unpulsed or ChgA 358–375 or RegII 48–64-pulsed NOD APCs with BDC2.5 TscFv was routinely performed for each newly produced batch. One of the batches was also tested on ChgA 29–42-pulsed NOD APCs (A). Additionally, S9/P2 TscFv was tested on NOD APCs pulsed with ChgA 29–42 and 358–371 and failed to recognize these, whereas APCs pulsed with RegII 48–64 stained positive with S9/P2 (Fig. S3, File S3). To determine if recombinant BDC2.5 TscFv could attenuate the stimulation of BDC2.5 TCR tg T-cells, spleen cells from BDC2.5 TCR tg mice were stimulated with mimotope RTRPLWVRME (3 µg/ml) in the presence of recombinant BDC2.5 or S9/P2 TscFv (10 µg/ml). TscFv BDC2.5, but not S9/P2 attenuated proliferation of BDC2.5 T-cells (D). Three experiments with different batches of BDC2.5 and S9/P2 were performed. The capacity of anti BDC2.5 antiserum to bind BDC2.5 TCR tg cells was assessed using PBMCs collected from BDC2.5 tg mice double-stained with anti CD4-PE and with serum from pre bleed or with anti BDC2.5 or S9/P2 TscFv immune serum. The protocol is depicted in (E) and the cells analyzed were in gate 1 (F). Antiserum from mice vaccinated with BDC2.5 TscFv (G), but not antiserum from mice vaccinated with S9/P2 TscFv (H) preferentially bound CD4^+^ T-cells from BDC2.5 TCR tg mice. PBMCs from three different BDC2.5 TCR transgenic mice were stained.

*Conversion of the BDC2.5 TCR into a TscFv retains its idiotype and vaccination with the TscFv yields anti BDC2.5 TCR specific serum.* TscFv BDC2.5 bound to NOD APCs pulsed with the peptide corresponding to the chromogranin A epitopes 29–42 and 358–371 (Fig. 6A and B), but failed to bind when the APCs were pulsed with Reg peptide 48–64 (Fig. 6C). In contrast TscFv S9/P2 failed to stain NOD APCs pulsed with the two ChgA epitopes (Fig. S3 in File S3). Furthermore, proliferation of BDC2.5 T-cells could be attenuated by addition of recombinant BDC2.5 TscFv but not by addition of S9/P2 TscFv (Fig. 6D) and anti BDC2.5 TscFv antiserum but not anti S9/P2 antiserum preferentially recognized CD4^+^ T-cells of BDC2.5 TCR transgenic mice (Fig. 6E, F, G and H).

### 2. Biological Effects of scFv Vaccination

#### 2.1 The NOD-SCID transfer model

*Diabetes transferred to NOD-SCID mice by BDC2.5 or NtfrRII specific CD4^+^ T-cells can be prevented by B but not T-cells from scFv-vaccinated donor mice if the specificity of the vaccine scFv and the specificity of the inducer cells are matched.*

The vaccine approach used here presupposes that B-cells (immunoglobulins) must play an important role in mediating the effects of the vaccination. Immunoglobulins can cross-react based on the structure of two similar idiotypes, which are built from different primary sequences (i.e. a library-derived scFv selected to have an idiotype similar to the actual TCR). To study clinical effects we first used an adoptive transfer model, which allowed us to investigate whether the effects depended on B- or T-cells. In addition, with this model the specificity of the vaccine scFv could be matched or mismatched with the specificity of the diabetogenic T-cells employed (the inducer T-cells). In the transfer model NOD-SCID recipients received test cells from NOD mice immunized with scFvs specific for I-A^g7^ NtfrRII peptide complexes or with BDC2.5 TscFv or cells from control-immunized mice. Subsequently they were injected with the diabetes inducer, NtfrRII specific or BDC2.5 T-cells. In the first experiment B-cells from mice immunized with BscFv clone C8 at 4 and 7 weeks of age were able to transfer protection against diabetes, while T-cells from C8-immunized and B-cells from non-immunized mice failed to do so (Table 2, experiment A). In a second experiment, B-cells from mice immunized at 8 and 11 weeks of age with BscFv clones C8 or D9 transferred protection from disease when induced by NtfrRII specific CD4^+^ T-cells but not when induced by BDC2.5 CD4^+^ T-cells. B-cells from donors immunized with the mutated form of D9 failed to protect from diabetes as did B-cells from mice immunized with Hen Egg Lysozyme (HEL) (Table 2, experiment B). In a third experiment B-cells but not T-cells from donors immunized with TscFv clone S9/P2 were able to transfer protection from T1D induced by NtfrRII specific CD4^+^ T-cells and diabetes induction by BDC2.5 T-cells was prevented by B but not T-cells from donors vaccinated with BDC2.5 TscFv (Table 2, experiment C). The results indicate that a) in this model B-cells from scFv-vaccinated donors are required for the clinical effects observed and b) protection from disease can be transferred only if the specificity of the vaccine scFv and the specificity of the inducer cell are matched (i.e. if the idiotypes of the inducer T-cells’ TCR and the vaccine scFv are matched).

**Table 2:** Adoptive transfer of type 1 diabetes to NOD-SCID recipients by diabetogenic CD4^+^ T-cells (inducer cells) can be prevented by B- but not T-cells (test cells) from donors immunized with scFvs if the specificity of the scFv and the inducer cell are matched.

Vaccination of test cell donors	Test cells transferred	Inducer cell specificity	# of recipients which developed T1D
**Exp A**			
BscFv clone C8	B-cells	NtfrRII	0/3
BscFv clone C8	T-cells	NtfrRII	3/3
None	B-cells	NtfrRII	3/3
**Exp B**			
BscFv clone C8	B-cells	NtfrRII	0/3
BscFv clone D9	B-cells	NtfrRII	0/3
BscFv clone D9	B-cells	BDC2.5	3/3
BscFv clone D9mut	B-cells	NtfrRII	3/3
HEL	B-cells	NtfrRII	3/3
**Exp C**			
TscFv clone S9/P2	B-cells	NtfrRII	0/6
TscFv clone S9/P2	T-cells	NtfrRII	6/6
TscFv clone BDC2.5	B-cells	BDC2.5	0/6
TscFv clone BDC2.5	T-cells	BDC2.5	3/3

Test cells were obtained from NOD mice immunized with the indicated scFvs at 4 and 7 weeks of age (experiment A and C) or at 8 and 11 weeks of age (experiment B). NtfrRII specific inducer cells were either obtained by depletion of B and CD8^+^ T-cells (A and C) or by negative isolation of CD4^+^ T-cells from NtfrRII-immunized mice (B). BDC2.5 inducer cells were obtained by negative isolation of CD4^+^ T-cells (B) or were non-separated spleen cells of BDC2.5 TCR tg mice (C). All mice in experimental groups with diabetes induced by NtfrRII specific T-cells had developed the disease by 82 days post transfer. Mice in groups free of diabetes were observed to 200 days post transfer. All mice in experimental groups with diabetes induced by BDC2.5 T-cells had developed the disease by 37 days post transfer. Mice in groups free of diabetes were observed to 100 days post transfer. B-cells were necessary to mediate prevention of disease and, for prevention to occur in this model, the specificity of the inducer cells has to match the specificity of the scFv used to vaccinate test cell donors.

#### 2.2 The BDC2.5 TCR transgenic NOD model

*T-cells in PBMCs of BDC2.5 TCR transgenic recipients of B-cells and antibodies from donors vaccinated with BDC2.5 TscFv undergo a gradual loss of responsiveness to mimotope stimulation accompanied by a loss of cytokine release.*

**Figure 7:**
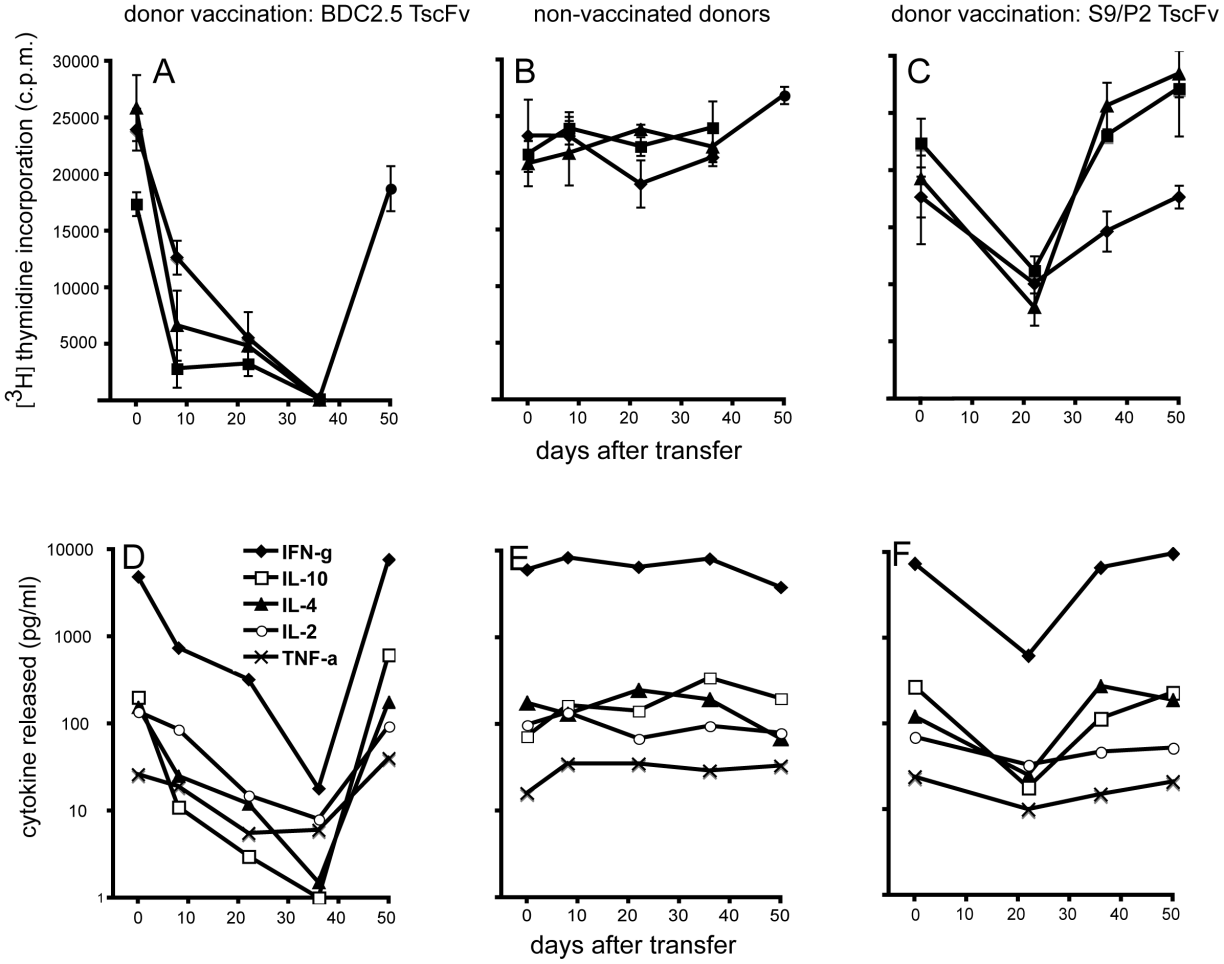
Effects in the BDC2.5 TCR transgenic NOD mouse (B-cells). To assess the effects B-cells derived from scFv-vaccinated mice would induce *in vivo* we transferred to BDC2.5 TCR tg recipients B-cells from NOD donors that had been vaccinated with TscFv BDC2.5 (A) or B-cells from non-vaccinated mice (B) or B-cells from donors that had been vaccinated with TscFv S9/P2 (C) (1.5×10^7^ in each case). At the indicated time points PBMCs were isolated and proliferative T-cell responses to the BDC2.5 mimotope (5 µg/ml) as well as cytokine-release in response to stimulation were measured. T-cell proliferation without antigen remained below 200cpm throughout and cytokine responses were at or below the detection threshold. Symbols in A-C represent data from individual mice, except at day 50 in A and B where PBMCs were pooled. Tissue culture supernatants from cells of individual mice were pooled for cytokine analysis (Bioplex). Transfer of B-cells from BDC2.5 TscFv-vaccinated donors induced an extended phase of anergy in CD4^+^ T-cells of BDC2.5 TCR tg recipients. This phase was characterized by a gradual loss of the proliferative response to mimotope stimulation and a corresponding loss of cytokine secretion in response to stimulation (A and D). BDC2.5 TCR tg recipients of B-cells from non-vaccinated donors failed to undergo this anergic phase (B and E) and recipients of B-cells from TscFv S9/P2-vaccinated donors exhibited a milder phase of shorter duration (C and F). Since the release curves for each measured cytokine were similar in shape we normalized them for statistical evaluation (value at day 0 = 100%). This allowed a comparison of the values obtained by different donor vaccinations at each time point beyond day 0. Statistically significant differences (t-test) were found at 8, 22 and 36 days between values in curves shown in D (BDC2.5-vaccinated donors) and E (non-vaccinated donors) and at 22 days between values in curves shown in E and F (S9/P2-vaccinated donors).

To study the mechanism by which diabetogenic T-cells react to vaccination with scFvs we used the BDC2.5 TCR transgenic NOD model. B-cells from non-vaccinated donors or from donors either vaccinated with S9/P2 or the BDC2.5 TscFv were transferred to BDC2.5 TCR transgenic recipients. We then tested the proliferative response of the T-cells in the recipients’ PBMCs to antigen (mimotope) stimulation. This allowed us to obtain a time course and a corresponding profile of the cytokine release from the stimulated T-cells. These profiles showed a marked decrease in antigen responsiveness and in the corresponding cytokine release that developed over a period of 5 weeks after transfer of donor B-cells if BDC2.5 TscFv was used to vaccinate donors (Fig. 7A and D). If donors were not vaccinated the T-cell response and cytokine release profiles in the recipients did not change (Fig. 7B and E). If donors were vaccinated with S9/P2 TscFv the T-cell response profile in the recipients failed to develop the profound anergy induced by B-cells from BDC2.5 TscFv-vaccinated donors but a milder reduction ensued (Fig. 7C and F). Loss of responsiveness was not attributable to a loss CD4^+^/Vβ4^+^ cells from the PBMCs of the recipients as the proportion of these cells remained unchanged throughout the duration of the experiment (Vβ4 is the β-chain of the BDC2.5 TCR). Furthermore, maximum peak intensity of FL-1 (anti Vβ4) in treated mice did not decrease between day 0 and day 36 post transfer but, on the contrary, showed a small increase indicating that no receptor downmodulation had taken place as consequence of the B-cell transfer (Table 3). The differences in the recipients’ T-cell response profiles to donor vaccination with either BDC2.5 or S9/P2 were not due to differences in the efficiency of vaccination or B-cell transfer as the antibody response to BDC2.5 or S9/P2 measured in the recipients’ serum were similar (Table 4).

**Table 3:** Proportion of CD4^+^/Vβ^+^ cells and peak fluorescence intensity of the Vβ4 signal in PBMCs of BDC2.5 TCR tg recipients after transfer of B-cells from donors vaccinated with TscFv BDC2.5.

Parameter [units]	Day 0	Day 36	Day 50
CD4^+^/Vβ4^+^ T-cells [% of total PBMCs]	0.78±0.17	0.66±0.25	1.17±0.4
Peak intensity Vβ4 [FL-1]	186±10	223±12	198±7

**Table 4:** Anti S9/P2 and anti BDC2.5 TscFv IgG in serum of BDC2.5 TCR tg recipients after transfer of B-cells from donors vaccinated with TscFv S9/P2 or BDC2.5.

Parameter [units]	Donor vaccination*	Day 22	Day 36	Day 50
anti BDC2.5 IgG [AU]	BDC2.5	76±20	81±53	77±25
anti S9/P2 IgG [AU]	S9/P2	55±23	77±56	99±23

* BDC2.5 TCR tg mice without transfer of B-cells from S9/P2 or BDC2.5-vaccinated donors have no detectable serum levels of anti S9/P2 or anti BDC2.5 IgG.

**Figure 8:**
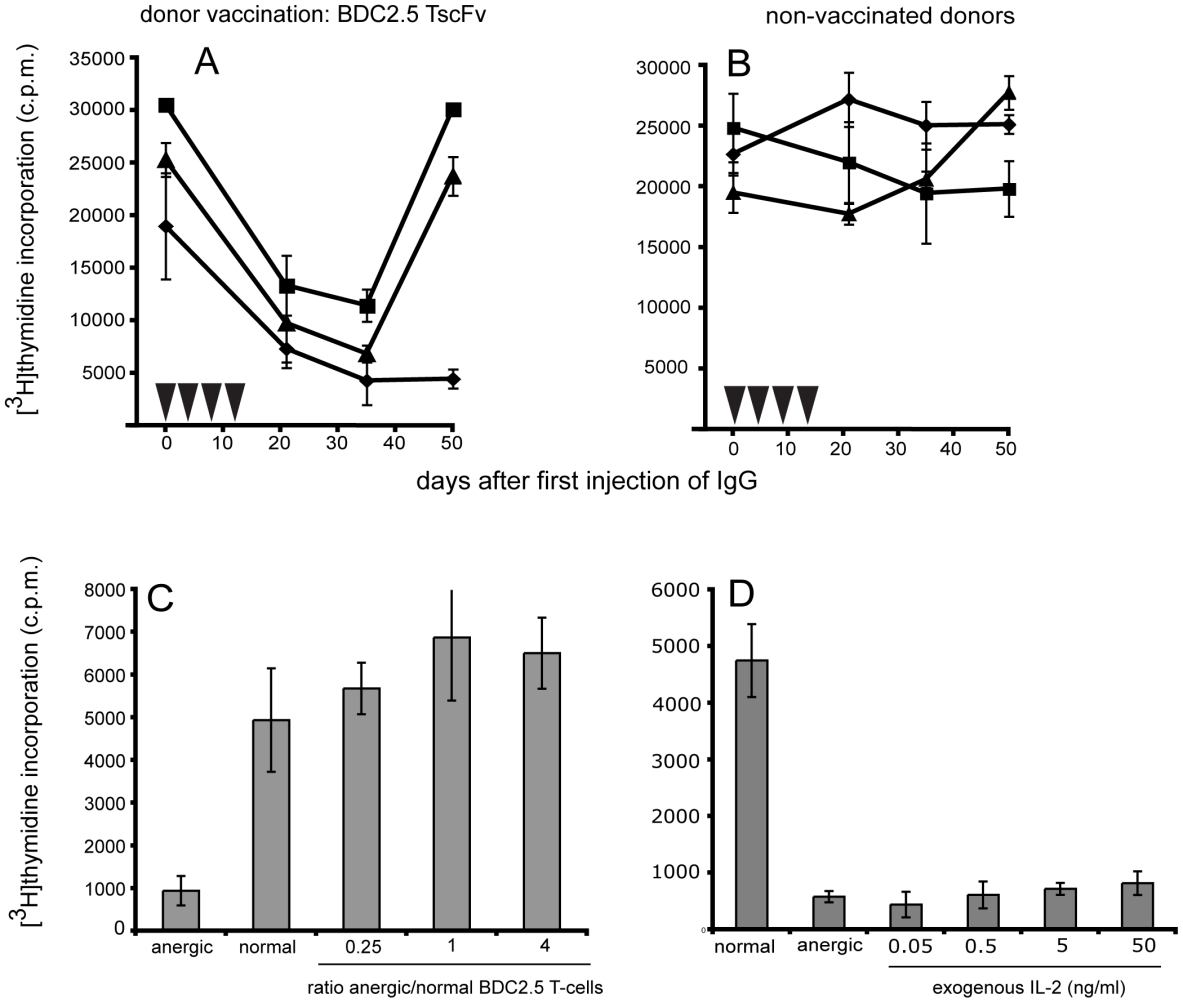
Effects in the BDC2.5 TCR transgenic NOD mouse (IgG). To confirm that immunoglobulin produced by B-cells from scFv-vaccinated mice induced the anergic phase seen after B-cell transfer, IgG from BDC2.5 TscFv-vaccinated donors (A) or IgG from non-vaccinated donors (B) was transferred to BDC2.5 TCR tg recipients. Both IgGs were purified by immobilized protein A/G and administered in 4 injections to BDC2.5 TCR tg recipients (▾, 3xi.v.; 1xi.p. 5 mg/kg each injection). To functionally characterize anergic T-cells we tested whether they were able to mediate ‘bystander’ suppression ( = attenuation of normal, non-anergic T-cells) and whether exogenous IL-2 restored their responsiveness. Anergic BDC2.5 T-cells generated *in vivo* were isolated from recipient’s PBMCs 4–5 weeks after the first transfer of anti BDC2.5 TscFv IgG. Before co-incubation with T-cells from untreated BDC2.5 TCR tg mice at the indicated ratios, anergic T-cells were irradiated (3000rads) (C). Anergic T-cells were incubated with IL-2 at the indicated concentration (D). The mimotope concentration for both experiments was 5 µg/ml (proliferative background response for both normal and anergic BDC2.5 TCR tg was <200 c.p.m). Anergic BDC2.5 T-cells did not suppress proliferation of normal, non-anergic BDC2.5 TCR tg T-cells and did not regain their proliferative response to mimotope stimulation upon addition of exogenous IL-2. Experiments C and D are representative graphs. Two independent experiments each for (C) and (D) were performed.

Since the results from both the NOD-SCID and the BDC2.5 TCR transgenic model indicated that B-cells from appropriately vaccinated donors were sufficient for the transfer of the clinical effect and the corresponding change in T-cell response profiles, we tested if injection of antibodies alone purified from BDC2.5 vaccinated mice could alter the response profile in the recipients. Repeated injections of IgG purified from the serum of BDC2.5 vaccinated NOD mice caused the T-cell response profile in the BDC2.5 TCR transgenic recipients to change in a similar manner to that observed after transfer of B-cells. Injection of IgG from non-immunized mouse serum did not alter the recipients’ T-cell response profile (Fig. 8A and B).

It is noteworthy, however, that the profound anergy of T-cells with the BDC2.5 TCR induced *in vivo* by B-cells and antibodies from donors vaccinated with BDC2.5 TscFv was not permanent. Seven weeks after transfer of B-cells, T-cell responsiveness was restored. This phenomenon did not correlate with a decrease in anti BDC2.5 or anti S9/P2 TscFv specific antibodies in the recipients of B-cells from scFv-vaccinated donors (Table 4).

Functional characterization of BDC2.5 T-cells in the anergic state showed that they did not inhibit proliferation of normal, non-anergic BDC2.5 cells and that exogenous IL-2 failed to recover the proliferative response to mimotope stimulation (Fig. 8C and D).

#### 2.3 Effects in NOD mice

*Vaccination with BDC2.5 or S9/P2 TscFv or with C8 BscFv delays diabetes in NOD mice.*

**Figure 9:**
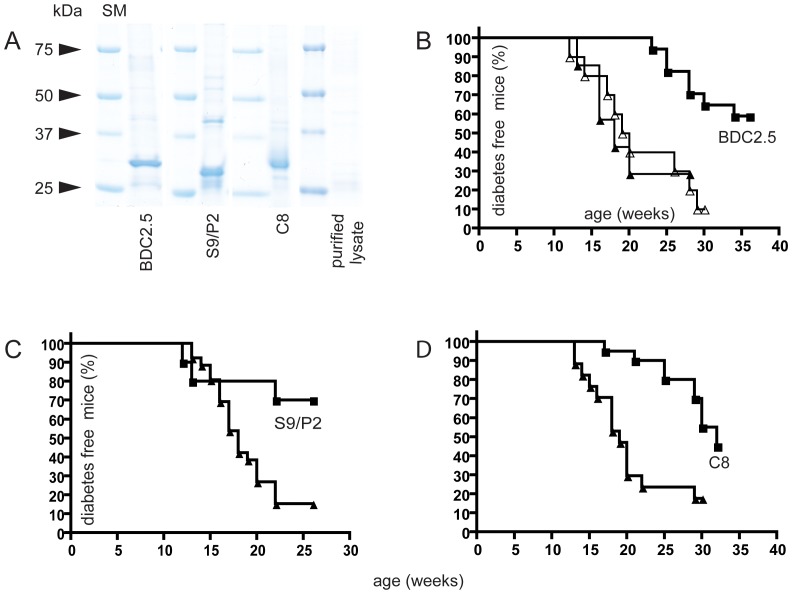
Effects in NOD mice. To investigate the effects of anti-idiotypic vaccination on the development of diabetes in NOD mice, recombinant scFvs were purified from bacterial lysates (*E. coli* strain HB2151) by Cobalt-NTA metal chelate affinity chromatography and analyzed by a Coomassie-stained SDS-PAGE gel (SM = size marker). In addition a lysate of non-transformed bacteria was produced and purified via metal chelate affinity chromatography as if it contained the recombinant protein (A). This preparation in alum served as one control, the other was alum alone. As shown in B, vaccination of NOD mice at 4 and 7 weeks of age with the purified control lysate (open triangles, n = 10) had no significant effect on diabetes onset as compared to vaccination with alum alone (closed triangles, n = 7). In contrast, vaccination with BDC2.5 TscFv (0.2 mg/kg; n = 17; squares) significantly delayed diabetes onset (P<0.001; logrank test). NOD mice vaccinated at 4 weeks and at 7 weeks of age with TscFv S9/P2 (0.2 mg/kg i.p.; n = 10; squares) exhibited a similar delay in diabetes onset as compared to control-vaccinated mice (alum, triangles, n = 26) (P<0.05) (C). Diabetes was also delayed by vaccination (at 4 weeks and at 7 weeks of age) with BscFv C8 (1.5 mg/kg i.p.; n = 17; squares) as compared to control vaccination (alum, triangles, n = 20, P<0.005) (D). Postponing initial vaccination with C8 to 10 weeks of age rendered the treatment ineffective.

The NOD-SCID and the BDC2.5 TCR transgenic adoptive transfer models demonstrate the clinical impact induced by attenuation of self-reactive CD4^+^ T-cells specific for one particular autoantigen, namely RegII (NtfrRII) or chromogranin A, and the specific role of B-cells in this process. However, the development of T1D in NOD mice (and humans) is mediated by self-reactive T-cells recognizing a variety of different autoantigens. To determine if a targeted approach could nevertheless influence the development of diabetes we immunized NOD mice with the recombinant purified scFvs (Fig. 9A). Vaccination with BDC2.5 TscFv significantly delayed T1D when compared with two different control groups, which had been immunized either with a purified lysate of non-transformed *E. coli* bacteria in alum or with alum alone (Fig. 9B). T1D could also be delayed by vaccination with two scFvs (S9/P2 and C8) specific for I-A^g7^ RegII peptide complexes (Fig. 9C and D). When the initial vaccination with C8 was postponed to 10 weeks of age, however, a significant delay of the disease could not be achieved (data not shown).

## Discussion

The current experimental approach is based on the assumption that an immune therapy targeting islet specific T-cells in T1D should originate from the MHC-peptide complex and the receptor recognizing it given that a) this is where the adaptive immune system generates specificity and b) the MHC is the element most profoundly affecting susceptibility to T1D.

For the acquisition of the vaccine antigen, we relied either on the TCR V-regions provided by an autoaggressive T-cell (BDC2.5) or on libraries of phage-displayed scFvs. BscFvs specific for MHC peptide complexes have previously been generated to selectively target malignant cells [28]–[31], as well as to visualize and quantify pMHC complexes in multiple sclerosis lesions [32]. Individual TscFvs (also known as single chain TCR) have also been generated [33]–[39]. However, TscFv libraries from lymphoid tissue have thus far not been produced and selected. For this reason we initially focused on the generation and selection of BscFv libraries supported by existing documentation and subsequently applied the expertise gained to the development of TscFv libraries based on our hypothesis that both types of scFv should be able to provide vaccine scFvs since both types could provide idiotypes recognizing ‘diabetogenic’ pMHC complexes. An MHC-peptide complex can be recognized either by an antibody or a T-cell receptor [40]–[42]. TCRs share a binding mode similar to antibody Fab fragments and, as shown by the comparison of their structures, two idiotypes that bind the same target structure can be closely related irrespective of their primary sequence [43]. The structural relatedness allows an immune serum that has been produced against one idiotype to cross-react with the other. Thus we undertook the isolation of TscFv clone S9/P2 by precipitation with anti D9 (BscFv) immune serum. D9 and S9/P2 recognized the same NtfrRII-derived peptide when presented in the context of I-A^g7^ and, despite the absence of extensive primary sequence homologies, were closely related structurally. Thus the TCR of an actual T-cell that recognizes RegII peptide 48–64 in context of I-A^g7^ is also likely to have an idiotype closely related to the one of S9/P2 and D9, allowing an anti D9 or S9/P2 idiotypic serum to cross-react with it. Consequently, the clinical effect of the immune response generated by this type of anti-idiotypic vaccination should rely on immunoglobulins since immunoglobulins can cross-react based on structural similarities between two antigens. This, in our view, is consistent with the observations that B-cells, but not T-cells were able to mediate diabetes prevention in the NOD-SCID transfer model and that the effect depended on a match between the specificity of the inducer cell and the vaccine scFv. Furthermore, B-cells and IgG from BDC2.5 scFv-vaccinated donors imparted a state of non-responsiveness to T-cells of BDC2.5 TCR tg recipients. Induction of anergy by anti-idiotypic IgG produced by the donor B-cells might thus be a mechanism explaining the disease preventive effect.

Anergy induction by anti-clonotypic antibodies in mouse and human T-cells has been studied *in vitro* for some time [44]; [45] and some features reported in those studies are consistent with those observed here, including the suppression of antigen-induced cytokine release, the lack of down-regulation of the targeted TCR, or loss of T-cells due to cell death or apoptosis. However, the non-responsiveness of anergic BDC2.5 T-cells to exogenous IL-2 and their inability to mediate suppression of normal BDC2.5 T-cells are features, which differ from those found in the earlier studies. The lack of *in vitro* suppression of non-anergic by anergic BDC2.5 TCR tg T-cells suggests that ‘bystander suppression’ is unlikely to be responsible for the clinical effects observed. If it were, then T-cells from the scFv-vaccinated donors would have caused diabetes suppression in the NOD-SCID transfer model. A similar argument might be made for the case where bystander suppression is replaced by regulatory T-cells. If these T-cells had been formed in the donors subsequent to scFv vaccination then - assuming that they were present in the spleen - they should have transferred ‘regulation/suppression’ to the recipients. This interpretation is also supported by a preliminary analysis of anergy-related gene-expression in normal vs. anergic BDC2.5 TCR tg cells, which did not exhibit upregulation of FoxP3 in anergic T-cells. Thus a model where exposure to anti-clonotypic antibodies ‘converts’ an effector cell into a T regulatory cell that suppresses effector cells of the same or different specificity is not consistent with our findings. Instead our data suggest that anti-idiotypic antibodies lead to phenotypic changes in the targeted effector cell such that it becomes anergic. The milder and shorter phase of reduced responsiveness observed in BDC2.5 TCR tg recipients of B-cells from S9/P2-vaccinated donors thus might be explained by residual cross-reactivity that allows anti S9/P2 clonotypic antibodies to weakly interact with the BDC2.5 TCR. This observation is consistent with the hypothesis that the antibody response against a given idiotype will not only recognize this particular idiotype but that there will be cross-reactivity with structurally related idiotypes. The cross-reactivity will be strong for closely related idiotypes and will become weaker as the structural distance (expressed, for example, in TM values) from the vaccine idiotype increases. Thus the target of an anti-idiotypic B-cell response is not a single idiotype but a group of structurally related idiotypes. This can explain the observation that precipitation of the TscFv library generated from pancreatic lymph nodes with anti D9 antiserum yielded not only a single clone, but a group of TscFvs that contained S9/P2. Although we have only modeled one member of this group our prediction is that the other members in this group are structurally closely related to S9/P2. Taken in the context of the pathogenesis of T1D this interpretation implies that vaccination with BDC2.5 or S9/P2 TscFv does not only affect chromogranin A or NtfrRII specific T-cells, but to a degree also T-cells with other specificities (TCR idiotypes). The degree to which a given idiotype is affected depends on its structural distance from the vaccine idiotype. Therefore the clinical effects induced by anti-idiotypic vaccinations are not the consequence of targeting only one single idiotype, but may be the result of targeting a group of idiotypes with the strongest effects centered on idiotypes closely related to the vaccine idiotype and with effects tapering off as structural distance from the vaccine idiotype increases. This interpretation may explain the observed clinical effect of anti-idiotypic vaccination in an autoimmune disease such as T1D with involvement of multiple idiotypes and indicates a mechanism by which the balance between specificity and efficacy of this approach might be manipulated. For example, the established TscFv base library could be reselected for clones recognizing I-A^g7^ complexed with epitopes of other autoantigens such as insulin and it could be tested whether vaccination with these clones alone or in combination with clones of different specificity or with entire selected libraries is more effective. In accordance with our definition of an ‘idiotype’ we view the TCR repertoire in islet infiltrates as a phenogram obtained by the conversion of a structural similarity matrix consisting of TM values (such as given in Table 1). From the results obtained here, this ‘structure-based’ phenogram will differ from the corresponding ‘primary sequence-based’ phenogram. However, as with the latter it will have branches and clusters and may form the basis for developing an annotated map of the local idiotype repertoire. Such a map may then guide the selection of vaccine idiotypes.

Given its potential clinical relevance we examined the temporal dynamics of the anergy as it develops *in vivo*. The rebound of responsiveness of the BDC2.5 TCR tg T-cells that occurred between 36 and 50 days after transfer of B-cells from BDC2.5 TscFv-vaccinated donors was unexpected, particularly since the rebound occurred without a decline in the serum levels of antibodies produced by the transferred B-cells. It is not clear whether the BDC2.5 TCR tg T-cells at day 50 (after the rebound) are the same in terms of their phenotype as those at day 0 or if the same time window applies to other lymphoid compartments, such as pancreatic lymph nodes. It is likely that the anergic state of the T-cells present in the PBMCs of BDC2.5 TCR tg recipients between day 0 and 50 post B-cell transfer is part of a more complex process of longer duration. Its duration appears to be too short to fully account for the protection in the NOD-SCID transfer model or the T1D delay in the scFv-vaccinated animals. More studies will be required to answer these questions.

In keeping with our anti-idiotypic approach targeting a restricted set of TCRs, vaccination of NOD mice with a BscFv specific for an I-A^g7^ NtfrRII peptide complex is only effective when treatment is started relatively early in the disease process. This finding may be explained by the fact that the profile of the NtfrRII specific T-cell response in the pancreatic lymph nodes has a first peak at 8 weeks of age and therefore anti-idiotypic immune intervention against NtfrRII-specific T-cells initiated at 10 weeks of age is too late to affect development of the disease. On the other hand induction of an anti-idiotypic immune response is effective when vaccination is timed to appropriately take into account the course of the spontaneous NtfrRII T-cell response in NOD mice. This view is supported by data showing that the age of the donors at which they receive the first vaccination does not influence efficacy in the NOD-SCID transfer model. Here coordination between the anti-idiotypic effect and the targeted inducer cells is maintained by the experimental design and consequently transfer of B-cells from donors with vaccination at either 4 weeks (experiment A, Table 2) or at 8 weeks of age (experiment B, Table 2) can achieve the same degree of disease prevention.

The translation of any ‘anti-idiotypic’ vaccine approach to humans would require the development of a personalized vaccine, or a vaccine suitable only for patients sharing a certain MHC allele. However, with an increasing number of human MHC alleles associated with elevated risk of T1D becoming available as transgenes, murine antigen presenting cells might allow the selection of phage-displayed single chain antigen receptor libraries. Furthermore, idiotype repertoires that would be selected on human pMHC complexes (Fig. 1) may not need to be generated from human V-regions. Here, we have provided evidence that an idiotype can be a useful vaccine regardless of its origin if it binds a relevant pMHC complex. Therefore, scFvs derived from murine libraries might be useful vaccines for humans provided they fulfill this condition.

## Supporting Information

File S1 Features of phage-display using the pAK system and procedure for library selection. M13 bacteriophage used for protein-display (A). Each phage displays a V-region single chain clone (an scFv representing an idiotype) fused to the truncated phage proteinIII (pIII). Each phage displays about 3–6 copies of the scFv-pIII fusion protein. Inside the phage cylinder is a DNA single strand. This strand contains the gene encoding the scFv-pIII fusion protein displayed on the phage head as well as a gene encoding chloramphenicol resistance. Thus the phage serves as link between the genetic information for a given scFv (idiotype) and the protein encoded by this information. Each phage can infect a bacterium, which will grow in chloramphenicol containing media. Dependent on the strain, infected bacteria can either produce the recombinant scFv or -after infection with a helper phage ( = rescue) - more scFv-displaying phages. These can be readily purified and concentrated from supernatant by precipitation with a polyethylene glycol 8000/NaCl solution. The phage coat is built from ≈2700 copies of protein VIII. This enhances staining efficiency when phage-displayed scFv are used as primary antibodies with an anti coat antibody as secondary. The scFv-pIII fusion protein is subject to gradual proteolysis and scFv displaying phages need to be prepared freshly for staining and selection experiments. Phages kept at 4°C in PBS will remain infective for extended periods of time (weeks). Phages are also resistant to extremes of pH and retain their infectivity after exposure to a pH range of 2–12. This allows the elution of bound phages by low or high pH during the process of library selection [18]; [19]. In phage-display methodology, specific binders are amplified over several selection rounds and it is therefore suitable for enriching clones with desired specificities provided the appropriate selection process has been applied to a non-selected library. An scFv library that has been subjected to a selection procedure is termed here a selected library. Clones of a selected library are tested individually to identify those with the desired specificity. The protocol depicted in (B) represents the selection process applied and shows a selection round (SN = supernatant). Spleen cells are pulsed (the antigen is added to cultured spleen cells) with the desired antigen (in this case the N-terminal fragment of RegII). This leads to the presence of pMHC complexes on the surface of the spleen cells, which serve as substrate to select the phage-displayed scFvs (idiotypes). The negative selection step on unpulsed spleen cells is added to reduce the presence of non-specific binders remaining after the positive selection step. IFN-γ is included in the incubation medium for spleen cells to enhance expression of I-A^g7^ and thus increase the number of ‘target’ pMHC complexes.
(TIF)

File S2 Steps and reagents involved in cloning of mouse TscFv libraries. Primers are given in Table S1–S5. PCR conditions were adopted from Krebber et al [16]. (SOE = splice by overlap extension). A C-terminal c-myc or 6xHis-tag is provided by the pAK system.
(TIF)

File S3 Staining of NOD APCs with S9/P2 TscFv. NOD APCs were pulsed with ChgA 29–42 or with ChgA 351–372 or with RegII 48–64. They were then stained with TscFv S9/P2. In contrast to BDC2.5 TscFv, S9/P2 did not recognize ChgA 29–42 or ChgA 351–372-pulsed NOD APCs. However, RegII 48–64-pulsed APCs were recognized by S9/P2.
(TIF)

File S4 Primers for TscFv library generation; peptides used in this study; sequences of scFv clones D9, C8 and S9/P2; sequence alignment between D9 and S9/P2.
(DOCX)
