# Supplementary figures and images for: Vaccination with Single Chain Antigen Receptors for Islet-Derived Peptides Presented on I-Ag7 Delays Diabetes in NOD Mice by Inducing Anergy in Self-Reactive T-Cells
Source: PLoS One. 2013 Jul 24;8(7):e69464. doi: 10.1371/journal.pone.0069464 (PMC3722102; doi:10.1371/journal.pone.0069464)

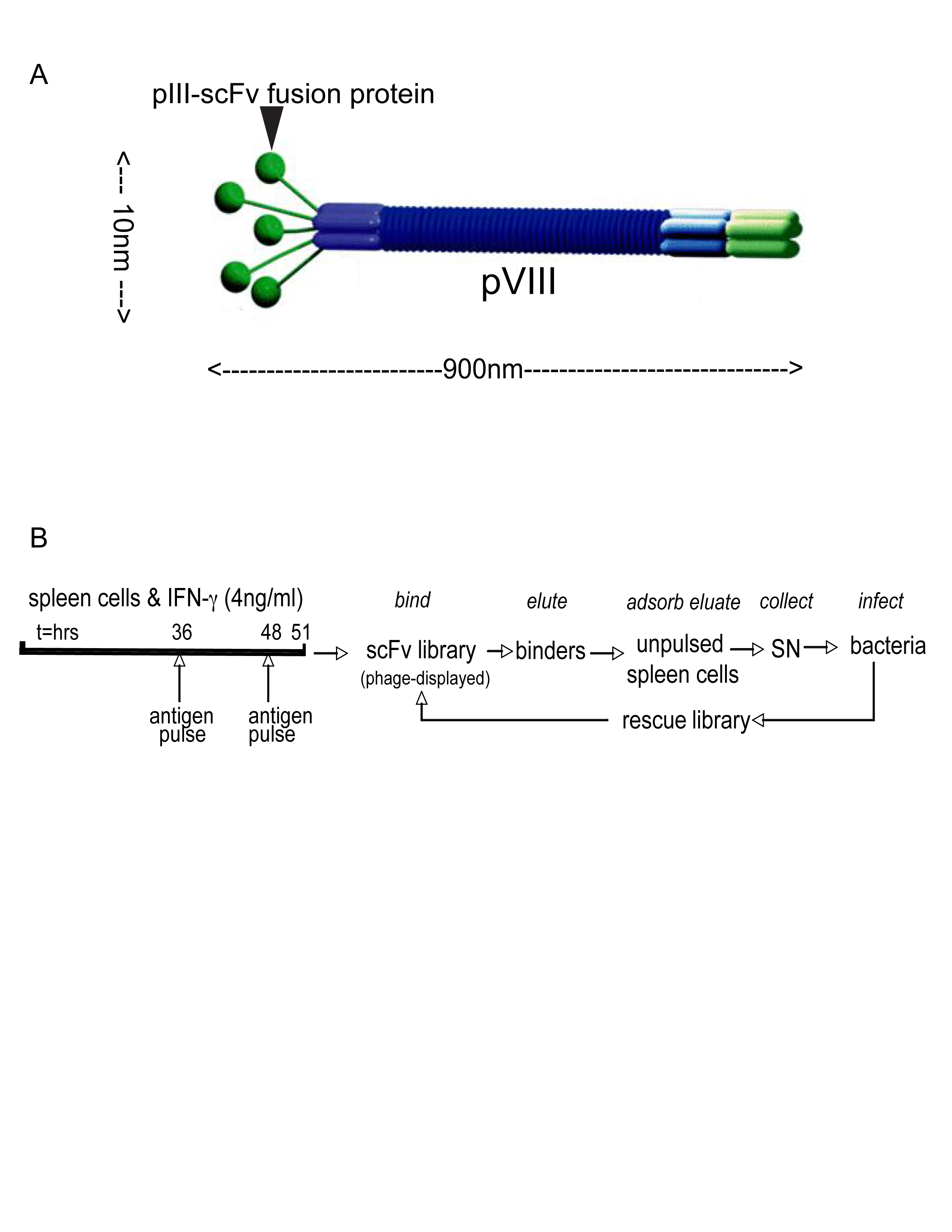

Supplement: File S1 — Features of phage-display using the pAK system and procedure for library selection. M13 bacteriophage used for protein-display (A). Each phage displays a V-region single chain clone (an scFv representing an idiotype) fused to the truncated phage proteinIII (pIII). Each phage displays about 3–6 copies of the scFv-pIII fusion protein. Inside the phage cylinder is a DNA single strand. This strand contains the gene encoding the scFv-pIII fusion protein displayed on the phage head as well as a gene encoding chloramphenicol resistance. Thus the phage serves as link between the genetic information for a given scFv (idiotype) and the protein encoded by this information. Each phage can infect a bacterium, which will grow in chloramphenicol containing media. Dependent on the strain, infected bacteria can either produce the recombinant scFv or -after infection with a helper phage ( = rescue) - more scFv-displaying phages. These can be readily purified and concentrated from supernatant by precipitation with a polyethylene glycol 8000/NaCl solution. The phage coat is built from ≈2700 copies of protein VIII. This enhances staining efficiency when phage-displayed scFv are used as primary antibodies with an anti coat antibody as secondary. The scFv-pIII fusion protein is subject to gradual proteolysis and scFv displaying phages need to be prepared freshly for staining and selection experiments. Phages kept at 4°C in PBS will remain infective for extended periods of time (weeks). Phages are also resistant to extremes of pH and retain their infectivity after exposure to a pH range of 2–12. This allows the elution of bound phages by low or high pH during the process of library selection [18]; [19]. In phage-display methodology, specific binders are amplified over several selection rounds and it is therefore suitable for enriching clones with desired specificities provided the appropriate selection process has been applied to a non-selected library. An scFv library that has been subjec [file pone.0069464.s001.tif]

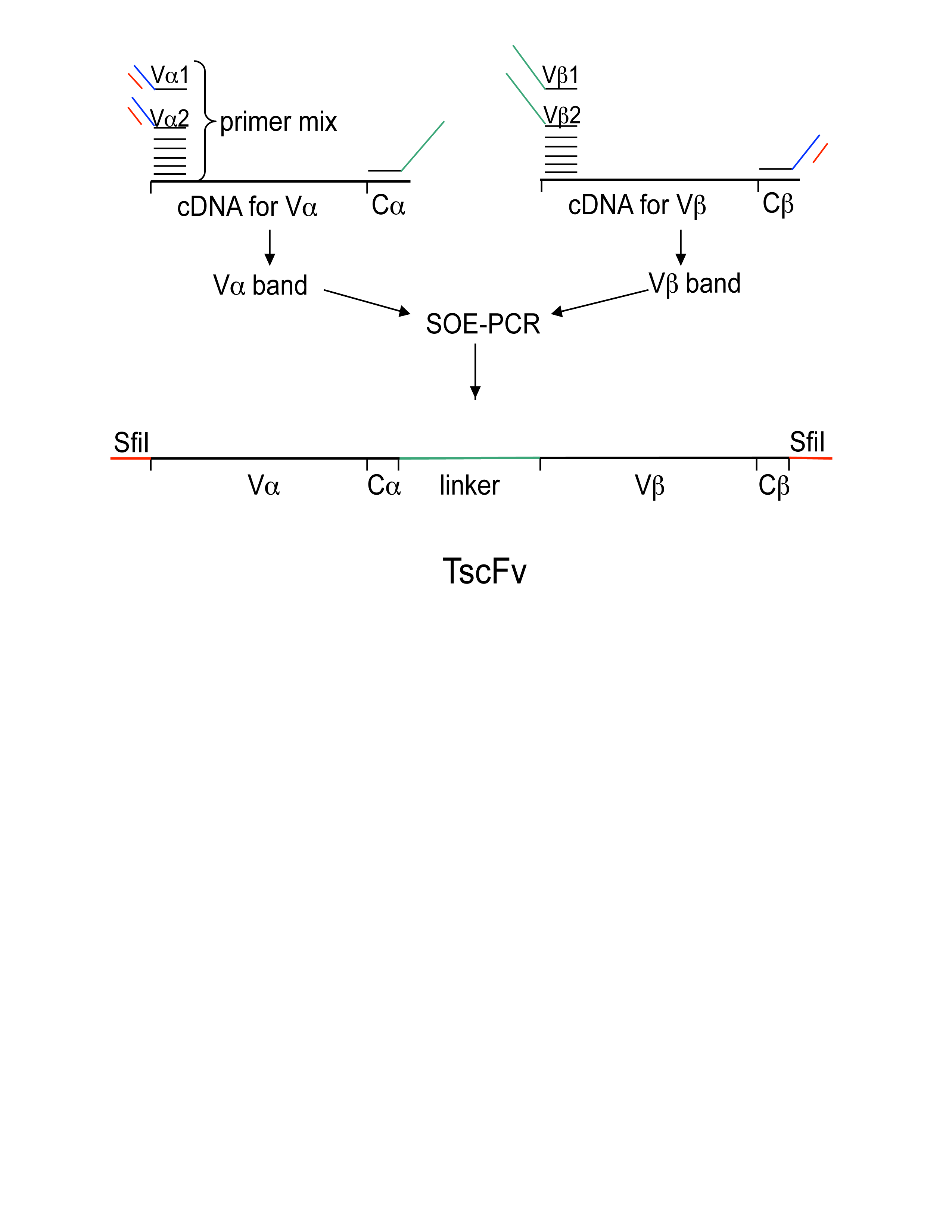

Supplement: File S2 — Steps and reagents involved in cloning of mouse TscFv libraries. Primers are given in Table S1–S5. PCR conditions were adopted from Krebber et al [16]. (SOE = splice by overlap extension). A C-terminal c-myc or 6xHis-tag is provided by the pAK system. (TIF) [file pone.0069464.s002.tif]

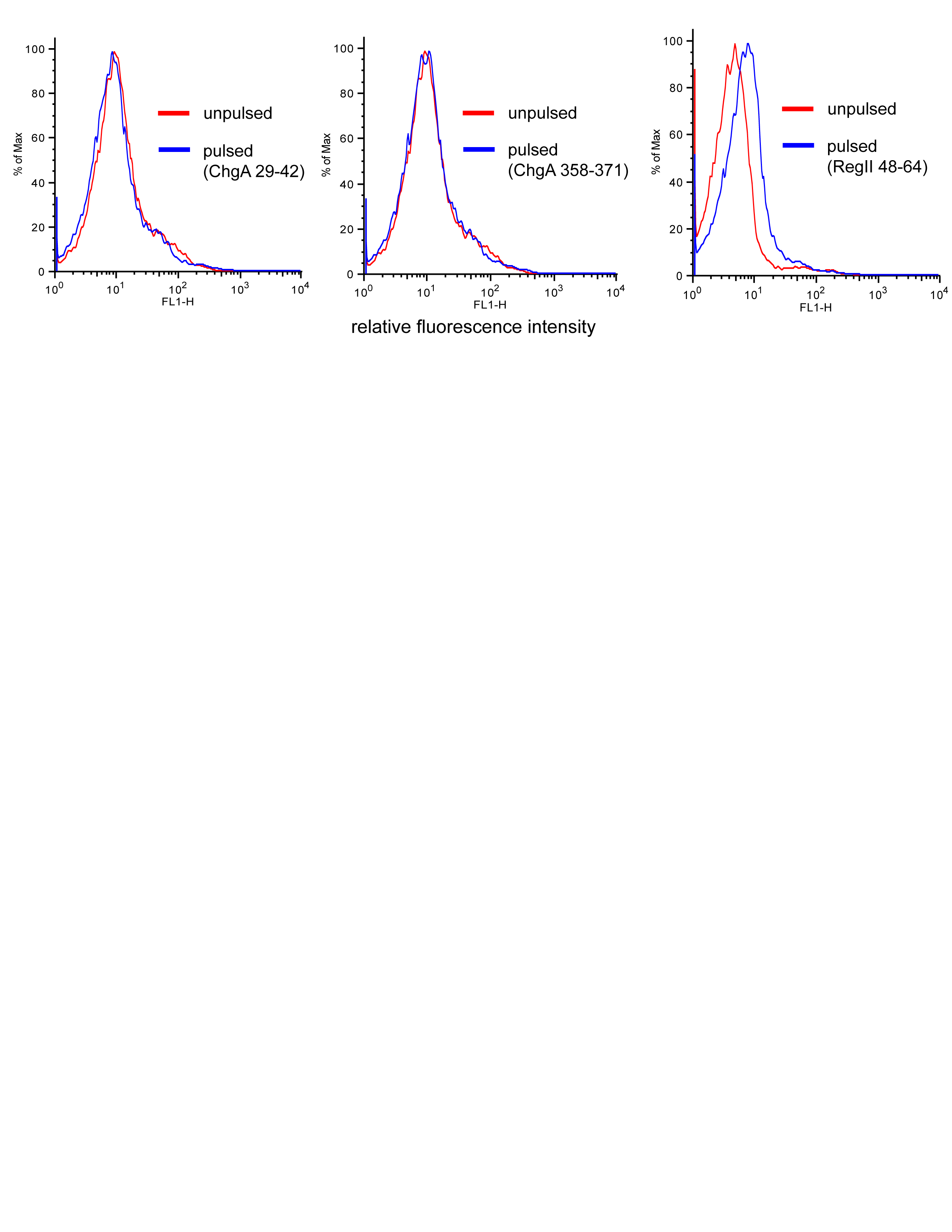

Supplement: File S3 — Staining of NOD APCs with S9/P2 TscFv. NOD APCs were pulsed with ChgA 29–42 or with ChgA 351–372 or with RegII 48–64. They were then stained with TscFv S9/P2. In contrast to BDC2.5 TscFv, S9/P2 did not recognize ChgA 29–42 or ChgA 351–372-pulsed NOD APCs. However, RegII 48–64-pulsed APCs were recognized by S9/P2. (TIF) [file pone.0069464.s003.tif]
