# Supplementary material for: Vaccination with Single Chain Antigen Receptors for Islet-Derived Peptides Presented on I-Ag7 Delays Diabetes in NOD Mice by Inducing Anergy in Self-Reactive T-Cells
Source: PLoS One. 2013 Jul 24;8(7):e69464. doi: 10.1371/journal.pone.0069464 (PMC3722102; doi:10.1371/journal.pone.0069464)
Supplement: File S4 — Primers for TscFv library generation; peptides used in this study; sequences of scFv clones D9, C8 and S9/P2; sequence alignment between D9 and S9/P2. (DOCX) [file pone.0069464.s004.docx]

**Table S1**

N-terminal primers used for cloning of the α chain of TscFv libraries

| NAME | SEQUENCE (5’to 3’) | d |
| --- | --- | --- |
| VA1 | CAR CAG AAK GTG CAG CAG AGC | 4 |
| VA1.5 | CAG GAG AAG GTA CAG CAG AGC | 1 |
| VA2 | CAG CAG MAG GAG AAG MRT GAC | 8 |
| VA3 | CAG ACA GTT TCC CAG TCT GAT G | 1 |
| VA3.1 | CAG TCA GTG ACR CAG CCC GAT G | 2 |
| VA4/VA8 | GAC TCH GTG ACY CAG ACR GAA G | 12 |
| VA4.1 | AAT TCA GTG ACC CAG ATG CAA G | 1 |
| VA4.2 | GAC TCA GTR AYC CAG ATG CAA G | 4 |
| VA4.3 | GAT TCC GTG ACT CAA ACA GAA G | 1 |
| VA5 | GAG CAG GTG GAG CAG CGC CC | 1 |
| VA6 | GAT GCT AAG ACT ACA CAA CC | 1 |
| VAD7 | SAG AAA GTG ATT CAG GTC TGG | 2 |
| VA9 | CAG TCC GTG GMM CAG CCT GAT G | 4 |
| VA10 | CAG CAA GTK CAG CAG AGY CC | 4 |
| VA11 | GAT MAG GTG RAG CAR AGT SC | 16 |
| VA12 | CTT GCC AAG ACC ACC CAG CC | 1 |
| VA13 | GAG CAG GTG GAG CAG CTT CC | 1 |
| VA14 | CAA GTG GAG CAG AGT CCT CAG | 1 |
| VA14.1 | AAG ACC CAA GTG GAG CAG AG | 1 |
| VA15 | GAG AAG GTC GAG CAA CAC GAG | 1 |
| VA16 | AAC ACT GTA GTG CAG AGC CC | 1 |
| VA17 | CAG AAG GTA ACA CAG ACT CAG | 1 |
| VA18 | CAA CTA GCA GAA GAG AAT CTG | 1 |
| VA19 | CAG GGT GTG GAG CAG CCT GC | 1 |
| VA20 | CAA CAG AAG ACT GGT GGC CAG | 1 |
|  |  |  |
| 5’ tail | **gcc atg gcg gac tac aca** |  |

All primers require the 5’tail given in the last row of the table in order to allow cloning into pAK. d=degree of degeneracy of a primer. Primer sequences were designed using data provided by Arden et al. [17].

**Table S2**

C-terminal primer used for cloning of the α-chain of TscFv libraries

| CA | 5’ AGC AGG TTC TGG GTT CTG GAT 3’ |
| --- | --- |
| 5’ tail | ***gga gcc gcc gcc gcc* aga acc acc acc acc *aga acc acc acc acc*** |

Capital letters indicate the part of the primer that anneals to the α-chain immediately adjacent to the end of the Vα region. Letters in lower caps denote the 5’tail necessary for introduction of one part of the linker during the splice by overlap extension PCR (SOE-PCR). Italics mark the region of the primer that anneals to the tail of the N-terminal β-chain primers during SOE-PCR.

**Table S3**

N-terminal primer used for cloning of the β-chain of TscFv libraries

| NAME | SEQUENCE (5’to 3’) | d |
| --- | --- | --- |
| VB1 | AAA ATT ACT CAG TCA CCA AG | 1 |
| VB2 | TTG CTG GAG CAA AAC CCA AGG | 1 |
| VB3 | AAA GTC ATT CAG ACT CCA AG | 1 |
| VB4 | AAA ATT ATC CAG AAA CCA AAA | 1 |
| VB5 | GGG GTT GTC CAG TCT CCA AG | 1 |
| VB6 | ATC ATT ACT CAG ACA CCC AAA | 1 |
| VB7 | AAA GTA ACC CAG ATG TCA AG | 1 |
| VB8 | GCA GTC ACC CAA AGY CCY AG | 4 |
| VB9 | ACG GTT AAG CAG AAC CCA AG | 1 |
| VB10 | GCT GTT TTC CAG ACT CCA AAC | 1 |
| VB11 | GGT GTC ATC CAA ACA CCT AGG | 1 |
| VB13 | GGA GTC ACC CAG TCT CCC AG | 1 |
| VB14 | ACT ATC CAT CAA TGG CCA GTT G | 1 |
| VB15 | CTC GTC TAT CAA TAT CCC AG | 1 |
| VB16 | AAA GTC TTA CAG ATC CCA AG | 1 |
| VB17 | GGA GTA ACC CAG ACT CCA CG | 1 |
| VB18 | CTC CTC TAC CAA AAG CCA AAC | 1 |
| VB19 | GGG GTT GTC CAG AAT CCT AG | 1 |
| VB20 | GCA GTT ACA CAG AAG CCA AG | 1 |
|  |  |  |
| 5’ tail | ***ggc ggc ggc ggc tcc* ggt ggt ggt gga tcc** |  |

All primers require the 5’tail given in the last row of the table in order to complete introduction of the linker between α and β chain. Italics mark the region of the primer that anneals to the tail of the C-terminal α-chain primer during SOE-PCR. Primer sequences were designed using data provided by Arden et al. [17].

**Table S4**

C-terminal primer used for cloning of the β-chain of TscFv libraries

| CB | 5’**g gaa ttc ggc ccc cga ggc** CAC ATT TCT CAG ATC CTC 3’ |
| --- | --- |

Capital letters indicate the part of the primer that anneals to the β-chain immediately adjacent to the end of the V-region. Letters in lower caps introduce the SfiI restriction site (underlined) at the C-terminal end of the assembled TscFv.

**Table S5**

Outer primers for amplification of the assembled TscFv

| NAME | SEQUENCE |
| --- | --- |
| O-N | 5’ **tta ctc gcg gcc cag ccg gcc atg gcg gac tac aca** 3’ |
| O-C | 5’ **g gaa ttc ggc ccc cga g** 3’ |

O-N introduces the SfiI site (underlined) at the N-terminal end of the assembled TscFv. SfiI is used by the pAK phagemid to directionally clone the scFv cassette.

**Table S6**

Peptide sequences of sets S and L covering the N-terminal fragment of RegII (NtfrRII)

Set S

| NtfrRII | 22-GQVAEEDFPLAEKDLPSAKINCPEGANAYGSYCYYLIEDRLTWGEADLFCQNMN-75 | |
| --- | --- | --- |
| Pool 1 | 23-QVAEEDFPLAEKDLPS-38 | 26-EEDFPLAEKDLPSAK-40 |
| Pool 2 | 28-DFPLAEKDLPSAKIN-42 | 30-PLAEKDLPSAKINCK-44 |
| Pool 3 | 32-NCKDLPSAKINCPEG-46 | 34-KDLPSAKINCPEGAN-48 |
| Pool 4 | 36-LPSAKINCPEGANAY-50 | 38-SAKINCPEGANAYGS-52 |
| Pool 5 | 40-KINCPEGANAYGSYP-54 | 42-NCPEGANAYGSYPEG-56 |
| Pool 6 | 44-PEGANAYGSYCYYLI-58 | 46-GANAYGSYCYYLIED-60 |
| Pool 7 | 48-NAYGSYCYYLIEDRL-62 | 50-YGSYCYYLIEDRLTW-64 |
| Pool 8 | 52-SYCYYLIEDRLTWGE-66 | 54-CYYLIEDRLTWGEAD-68 |
| Pool 9 | 56-YLIEDRLTWGEADLF-70 | 58-IEDRLTWGEADLFCQ-72 |
|  | 60-DRLTWGEADLFCQNMN-75 |  |

The top row gives the sequence of NtfrRII. Peptide pools 1 to 9 were used to pulse NOD APCs in order to determine the epitope recognized by D9.

Set L

| Peptide 1 (P1) | 22-GQVAEEDFPLAEKDLPSAKINC-43 |
| --- | --- |
| Peptide 2 (P2) | 34-KDLPSAKINCPEGANAYGSY-53 |
| Peptide 3 (P3) | 44-PEGANAYGSYCYYLIEDRLT-63 |
| Peptide 4 (P4) | 59-EDRLTWGEADLFCQNMN-75 |

Peptides P1-4 covering NtfrRII were used to determine whether the epitope recognized by D9 in the context of I-A^g7^ was able to activate autoaggressive T-cells.

Other peptides

|  | Antigen |  |
| --- | --- | --- |
| Peptide 35 | glutamic acid decarboxylase | 524-SRLSKVAPVIKARMMEYGTT-543 |
| ChgA epitope 1 | chromogranin A | 24-DTKVMKCVLEVISD-42 |
| ChgA epitope 2 | chromogranin A | 358-WSRMDQLAKELTAE-371 |
| Mimotope | --------- | RTRPLWVRME |

**Table S7**

DNA and deduced amino acid sequence of BscFv clone D9 (V_l_-linker-V_h_)

1 GAT ATT GTG ATG ACG CAG TCT CCA GCC ACC CTG TCT GTG ACT CCA GGA GAT AGA GTC TCT

**D I V M T Q S P A T L S V T P G D R V S**

61 CTT TCC TGC AGG GCC AGC CAG AGT ATT AGC GAC TAC TTA CAC TGG TAT CAA CAA AAA TCA

**L S C R A S Q S I S D Y L H W Y Q Q K S**

121 CAT GAG TCT CCA AGG CTT CTC ATC AAA TAT GCT TCC CAA TCC ATC TCT GGG ATC CCC TCC

**H E S P R L L I K Y A S Q S I S G I P S**

181 AGG TTC AGT GGC AGT GGA TCA GGG TCA GAT TTC ACT CTC AGT ATC AAC AGT GTG GAA CCT

**R F S G S G S G S D F T L S I N S V E P**

241 GAA GAT GTT GGA GTG TAT TAC TGT CAA AAT GGT CAC AGC TTT CCG TAC ACG TTC GGA GGG

**E D V G V Y Y C Q N G H S F P Y T F G G**

301 GGG ACC AAG TTG GAA ATA AAA CGT GGT GGT GGT GGT TCC GGC GGC GGC GGC TCC GGC GGC

**G T K L E I K R** *G G G G S G G G G S G G*

361 GGC GGC TCC GGT GGT GGT GGA TCC CAG GTC CTA CTG CAG CAG CCT GGG GCT GAG CTG GTG

*G G S G G G G S* **Q V L L Q Q P G A E L V**

421 AGG CCT GGG TCT TCA GTG AAG CTG TCC TGC AAG GCT TCT GGC TAC ACC TTC ACC AGC TAC

**R P G S S V K L S C K A S G Y T F T S Y**

481 TGG ATG CAT TGG GTG AAG CAG AGG CCT ATA CAA GGC CTT GAA CGG ATT GGT AAC ATT GAC

**W M H W V K Q R P I Q G L E R I G N I D**

541 CCT TCT GAT AGT GAA ACT CAC TAC AAT CAA AAG TTC AAG GAC AAG GCC ACA TTG ACT GTA

**P S D S E T H Y N Q K F K D K A T L T V**

601 GAC AAA TCC TCC AGC ACA GCC TAC ATG CAG CTC AGC AGC CTG ACA TCT GAG GAC TCT GCG

**D K S S S T A Y M Q L S S L T S E D S A**

661 GTC TAT TAC TGT GCA AGA TAC TAT AGT AAC TAC TGG TAC TTC GAT GTC TGG GGC ACA GGG

**V Y Y C A R Y Y S N Y W Y F D V W G T G**

721 ACC ACG GTC ACC GTT TCC TCG

**T T V T V S S**

The light and heavy chain amino acid sequence are printed on gray background. The linker is in italics and CDR3 are underlined. The sequence in blue has been deleted in D9mut. GeneBank accession number for D9 light chain KC335196; for D9 heavy chain: KC335197.

**Table S8**

DNA and deduced amino acid sequence of BscFv clone C8

1 GAC ATT CAG ATG ACG CAG TCT CCT GCC TCC CAG TCT GCA TCT CTG GGA GAA AGT GTC ACC

**D I Q M T Q S P A S Q S A S L G E S V T**

61 ATC ACA TGC CTG GCA AGT CAG ACC ATT GGT ACA TGG TTA GCA TGG TAT CAG CAG AAA CCA

**I T C L A S Q T I G T W L A W Y Q Q K P**

121 GGG AAA TCT CCT CAG CTC CTG ATT TAT GCT GCA ACC AGC TTG GCA GAT GGG GTC CCA TCA

**G K S P Q L L I Y A A T S L A D G V P S**

181 AGG TTC AGT GGT AGT GGA TCT GGC ACA AAA TTT TCT TTC AAG ATC AGC AGC CTA CAG GCT

**R F S G S G S G T K F S F K I S S L Q A**

241 GAA GAT TTT GTA AGT TAT TAC TGT CAA CAA CTT TAC AGT ACT CCG TAC ACG TTC GGA GGG

**E D F V S Y Y C Q Q L Y S T P Y T F G G**

301 GGG ACC AAG TTG GAA ATA AAA CGT GGT GGT GGT GGT TCT GGT GGT GGT GGT TCT GGC GGC

**G T K L E I K R** G G G G S G G G G S G G

361 GGC GGC TCC GGC GGC GGC GGC TCC GGT GGT GGT GGA TCC GAG GTC CAG CTG CAA CAG TCT

G G S G G G G S G G G G S **E V Q L Q Q S**

421 GGA CCT GTG CTG GTG AAG CCT GGG GCT TCA GTG AAG ATG TCC TGT AAG GCT TCT GGA TAC

**G P V L V K P G A S V K M S C K A S G Y**

481 ACA TTC ACT GAC TAC TAT GTA AAC TGG ATG AAA CAG AGC CAT GGA GAG AGC CTT GAG TGG

**T F T D Y Y V N W M K Q S H G E S L E W**

541 ATT GGA ATT ACT ATT CCT TAT AAC GGT AAT AGT AAT TAT AAC CAG AAA TTC AAG GGC AAG

**I G I T I P Y N G N S N Y N Q K F K G K**

601 GCC ACT TTG ACT GTT GAC AAG TCC TCC AAC ACA GCC TAC ATG GAG CTC AAT AGT CTG ACA

**A T L T V D K S S N T A Y M E L N S L T**

661 TCT GAG GAC TCT GCA GTC TAT TAT TGT GCA AGA GAG GGC GAC GTG GGG ATG GAC TAC TGG

**S E D S A V Y Y C A R E G D V G M D Y W**

721 GGT CAA GGA ACC TCA GTC ACC GTC TCC TCG

**G Q G T S V T V S S**

DNA and deduced amino acid sequence of BscFv clone C8 in the form V_l_-linker-V_h_. GeneBank accession number for C8 light chain KC335198; for C8 heavy chain: KC335199.

**Table S9**

DNA and deduced amino acid sequence of TscFv clone S9/P2 in the form Vα-linker-Vβ

1 GAT TCC GTG ACT CAA ACA GAA GGC CTG GTC ACT CTC ACA GAA GGG TTG CCT GTG ATG CTG

**D S V T Q T E G L V T L T E G L P V M L**

61 AAC TGC ACC TAT CAG ACT ATT TAC TCA AAT GCT TTC CTT TTC TGG TAT GTG CAC TAT CTC

**N C T Y Q T I Y S N A F L F W Y V H Y L**

121 AAT GAA TCC CCT CGG CTA CTC CTG CGG AGC TCC ACA GAC AAC AAG AGG ACC GAG CAC CAA

**N E S P R L L L R S S T D N K R T E H Q**

181 GGG TTC CAC GCC ACT CTC CAT AAG AGC AGC AGC TCC TTC CAT CTG CAG AAG TCC TCA GCG

**G F H A T L H K S S S S F H L Q K S S A**

241 CAG CTG TCA GAC TCT GGC CTG TAC TAC TGT GCT TTG AGT TAT CTA CCA GGC ACT GGG AGT

**Q L S D S G L Y Y C A L S Y L P G T G S**

301 AAC AGG CTC ACT TTT GGG AAA GGC ACC AAA TTC TCA CTC ATC CCG AAC ATC CAG AAC CCA

**N R L T F G K G T K F S L I P N I Q N P**

361 GAA CCT GCT GGT GGT GGT GGT TCT GGT GGT GGT GGT TCT GGC GGC GGC GGC TCC GGT GGT

**E P A** *G G G G S G G G G S G G G G S G G*

421 GGT GGA TCC TTG CTG GAG CAA AAC CCA AGG TGG CGT CTG GTA CCA CGT GGT CAA GCT GTG

*G G S* **L L E Q N P R W R L V P R G Q A V**

481 AAC CTA CGC TGC ATC TTG AAG AAT TCC CAG TAT CCC TGG ATG AGC TGG TAT CAG CAG GAT

**N L R C I L K N S Q Y P W M S W Y Q Q D**

541 CTC CAA AAG CAA CTA CAG TGG CTG TTC ACT CTG CGG AGT CCT GGG GAC AAA GAG GTC AAA

**L Q K Q L Q W L F T L R S P G D K E V K**

601 TCT CTT CCC GGT GCT GAT TAC CTG GCC ACA CGG GTC ACT GAT ACG GAG CTG AGG CTG CAA

**S L P G A D Y L A T R V T D T E L R L Q**

661 GTG GCC AAC ATG AGC CAG GGC AGA ACC TTG TAC TGC ACC TGC AGT GCA AGA CTG GGG GAT

**V A N M S Q G R T L Y C T C S A R L G D**

721 AAC CAA GAC ACC CAG TAC TTT GGG CCA GGC ACT CGG CTC CTC GTG TTA GAG GAT CTG AGA

**N Q D T Q Y F G P G T R L L V L** **E D L R**

781 AAT GTG

**N V**

The α and β chain amino acid sequences are printed on gray background. The amino acids - IQNPEPA - and - EDLRNV - belonging to constant regions adjacent to the V regions of the α or β chain respectively and therefore contained in every TscFv are printed on white background. The linker is in italics and CDR3 are underlined. GeneBank accession number for S9/P2 alpha chain KC335200; for S9/P2 beta chain: KC335201.

**Table S10**

Alignment of amino acid sequences of D9 (BscFv) and S9/P2 (TscFV) recognizing the I-A^g7^ NtfrRII (48-64) complex

| D9 V_l_ | **DIVMTQSPATLSVTPGDRVSLSCRASQSISD**.**YLHWYQQKSHESPRLLIKYASQSISGI** |
| --- | --- |
| S9/P2 Vα | .**DSVTQTEGLVTLTEGLPVMLNCTYLTIYSNAFLFWYVHYLNESPRLLLRSSTDNKRTE** |
| *consensus* | ....TQ.......T.G..V.L.C......S#.%L.WY.....ESPRLL.....#..... |
|  |  |
| D9 V_l_ | **PSRFSGS**..**GSGSDFTLSINSVEPEDVGVYYCQNGH**.....**SFPYTFGGGTKLEIKR**. |
| S9/P2 Vα | **HQGFHATLHKSSTSFHLQKSSAQLSDSGLYYCALSYLPGTGSNRLTFGKGTKFSLIPN** |
| *consensus* | ...F......S...F.L...S.#..D.G.YYC.........S...TFG.GTK...... |

| D9 V_H_ | **QVLLQQPGAELVRPGSSVKLSCKASGYTFTSYWMHWVKQRPIQGLERIGNI**.**DPSDSETHY** |
| --- | --- |
| S9/P2 Vβ | .**LLEQNPRWRLVPRGQAVNLRCILKNSQYP**..**WMSWYQQDLQKQLQWLFTLRSPGDKEVKS** |
| *consensu*s | ..L.Q#P...LV..G..V.L.C......%...WM.W..Q.....L#.......P.D.E... |
|  |  |
| D9 V_H_ | **NQKFKDKATLTVDKSSSTAYMQLSSLTSEDSAVYYCARYYSNYWYFDVWGTGTTVTVSS** |
| S9/P2 Vβ | **LPGADYLATRVTD**...**TELRLQVANMSQGRTLYCTCSARLGDNQDTQYFGPGTRLLVL**. |
| *consensus* | .......AT...D.......$Q...$.........C.....#....#..G.GT...V.. |

The linker and the α and β chain C-terminal residues (IQNPEPA and EDRLNV) common to all TscFvs have been omitted for this alignment (% is F or Y; $ is L or M, # is N, D, Q or E).
